# A metagenomic approach to characterize temperate bacteriophage populations from Cystic Fibrosis and non-Cystic Fibrosis bronchiectasis patients

**DOI:** 10.3389/fmicb.2015.00097

**Published:** 2015-02-18

**Authors:** Mohammad A. Tariq, Francesca L. C. Everest, Lauren A. Cowley, Anthony De Soyza, Giles S. Holt, Simon H. Bridge, Audrey Perry, John D. Perry, Stephen J. Bourke, Stephen P. Cummings, Clare V. Lanyon, Jeremy J. Barr, Darren L. Smith

**Affiliations:** ^1^Faculty of Health and Life Sciences, University of Northumbria at NewcastleNewcastle Upon Tyne, UK; ^2^Public Health EnglandLondon, UK; ^3^Freeman HospitalNewcastle Upon Tyne, UK; ^4^Institute of Cellular Medicine, Newcastle UniversityNewcastle Upon Tyne, UK; ^5^Royal Victoria InfirmaryNewcastle Upon Tyne, UK; ^6^Department of Biology, San Diego State UniversitySan Diego, CA, USA

**Keywords:** *Pseudomonas aeruginosa*, temperate bacteriophage, cystic fibrosis, non-Cystic fibrosis bronchiectasis, mixed phage populations

## Abstract

*Pseudomonas aeruginosa* (Pa), normally a soil commensal, is an important opportunistic pathogen in Cystic Fibrosis (CF) and non-Cystic Fibrosis Bronchiectasis (nCFBR). Persistent infection correlates with accelerated decline in lung function and early mortality. The horizontal transfer of DNA by temperate bacteriophages can add gene function and selective advantages to their bacterial host within the constrained environment of the lower lung. In this study, we chemically induce temperate bacteriophages from clonal cultures of Pa and identify their mixed viral communities employing metagenomic approaches. We compared 92 temperate phage metagenomes stratified from these clinical backgrounds (47 CF and 45 nCFBR Pa isolates) using MG-RAST and GeneWise2. KEGG analysis shows the complexity of temperate phage accessory gene carriage increases with duration and severity of the disease. Furthermore, we identify the presence of Ig-like motifs within phage structural genes linked to bacterial adhesion and carbohydrate binding including Big_2, He_Pig, and Fn3. This study provides the first clinical support to the proposed bacteriophage adherence to mucus (BAM) model and the evolution of phages interacting at these mucosal surfaces over time.

## Introduction

Chronic respiratory diseases are associated with about 4 million deaths globally per annum (WHO^1^). Cystic Fibrosis (CF) is a rare but well documented inherited chronic respiratory disease that is characterized by chronic bacterial infections of the lung (Mall and Boucher, 2014). Non-Cystic Fibrosis bronchiectasis (nCFBR) is usually associated with an older population; it is an abnormal and irreversible dilation of the lower bronchi. Unifying features between CF and nCFBR are the propensity for *Pseudomonas aeruginosa* (Pa) to be an opportunistic pathogen and abnormal mucus retention in the lower respiratory tract.

Pa is challenging to study at the genome level due to the presence of multiple genomic islands and a burgeoning accessory genome that may well correlate with its opportunistic nature. Opportunistic bacteria colonize the lungs of patients with chronic respiratory disease and utilize the nutrient rich mucus lining of the lower airways allowing for bacterial replication and evolution to occur in an often deteriorating microenvironment (Nelson et al., 2010; Hauser et al., 2011; Rudkjobing et al., 2011). Other bacteria that are commonly isolated from chronically infected lung include; *Staphylococcus aureus, Haemophilus. influenzae, Stenotrophomonas maltophilia, Achromobacter xylosoxidans*, whilst *Burkholderia cepacia* complex and Pa are descriptive of the CF lung and are linked to poor clinical outcomes including lowered lung function (Lipuma, 2010). Multiple phages have been previously identified in Pa isolated from the CF lungs (Winstanley et al., 2009).

Bacteriophages can be either classed as lytic or temperate. Lytic phages upon entry into their host bacterium rapidly propagate leading to cell lysis. Importantly, lytic phages do not become integrated into the bacterial chromosome; this is in contrast to temperate phages which upon entry into the cell integrate into the host genome as a prophage. Temperate phages infect their bacterial host and lay dormant within the bacterial host chromosome until they are induced from their host, forming an infective phage particle. It has been noted that phages typically outnumber bacteria by a factor of 10 (Fineran et al., 2009). Previous metagenomic studies focusing on viruses have identified novel patterns associated with evolution and novel viral particles (Kristensen et al., 2010). In this study, temperate bacteriophages were induced from their bacterial host using Norfloxacin (Matsushiro et al., 1999).

The major focus of numerous genome studies is determination of the core phage genome architecture. This study uses a metagenomic approach to elucidate the depth, function and complexity of phages evolving in a constrained environment of the lower lung. Conventional genome assembly tools try to compile mixed communities into single phages as they try to match and overlay similarity of sequence composition. Here we employ Metagenomics Rapid Annotations based on Subsystem Technology (MG-RAST) to overcome the need to assemble single phages and focus on the accessory genomes functionality. Another advantage of using MG-RAST is to generate Kyoto Encyclopedia of Genes and Genomes (KEGG) pathways; these allow for analysis of gene functionality via linking genetic information with higher order functional information (Kanehisa and Goto, 2000).

Here we focused on lysogenic phages, as they form an intrinsic part of the adaptation and evolution of bacteria (Bankevich et al., 2012). Temperate phages have been shown to carry a range of genes that can alter the fitness or pathogenicity of a bacterium. An example would be the ability to encode functional toxins in their host bacterium which in turn can increase the severity of disease and may influence its progression (Beddoe et al., 2010; Boyd et al., 2012; Dubreuil, 2012). Our focus was on phage accessory genes, which link temperate phages to bacterial adaptation and evolution in chronic lung infections. Phage accessory genes are understudied as they are small with no offered function; importantly these genes are usually shared between phages suggesting a conserved role in their biology or for subversion of their host (Smith et al., 2012). Here we compare clinical data and the complexity of phage-encoded accessory gene function to link to the pathophysiology of the chronic lungs in patients with CF and nCFBR. Metagenomic studies are beneficial for studying mixed viral communities as they utilize culture independent methods allowing for the observation of viral communities that lack a known propagating host and therefore, can be underrepresented in some studies. This is also a problem in bacterial studies with the inability to culture all strains in the laboratory so increasing the need for direct DNA sequencing methodologies that limit culture bias (Hugenholtz et al., 1998). Further complexity is added to mixed population genome assemblies as bacteria and viruses carry homologous genes with conserved order which can make them harder to separate bioinformatically (Drancourt et al., 2000; Boudewijns et al., 2006). Metagenomics can further be utilized to investigate pan-functionality in a sample compared to more traditional taxonomic approaches (Tringe et al., 2005), making it an ideal tool for *de novo* studies as it negates the need for previous knowledge of the sample (Roux et al., 2014).

Mucus forms the first line of defense for protection against pathogenic infection in the lung forming a physical barrier between the center of the airways and the underlying epithelial cells (Hansson, 2012; Barr et al., 2013). Mucus is composed mostly of mucin, a host produced glycoprotein but other macromolecules are also known to be present (Kim and Ho, 2010). The Bacteriophage Adherence to Mucus (BAM) model proposes that lytic phages adhere to carbohydrate residues present within the mucus layer, and provide a layer of immunity to incoming bacteria (Barr et al., 2013). The BAM model is mediated by structurally displayed carbohydrate-adherence domains, such as the immunoglobulin (Ig)–like domain present on the capsid of phage T4 (Hoc). Structural proteins with associated Ig-like domains have been found in approximately 25% of the sequenced dsDNA phages, demonstrating their ubiquity and their potential importance in aiding phage survival (Fraser et al., 2007). Such structural domains have been seen to be indispensable for phage propagation in laboratory settings as they mediate interactions between the phage and its host cell (McMahon et al., 2005; Fraser et al., 2007).

Here we investigate whether the temperate phages isolated from the mucus rich environments of CF and nCFBR patients' lungs support the BAM model and its clinical relevance. We utilize the BAM model to propose a different strategy for phages to disseminate across their host These observations led to the hypothesis that these domains may aid in both the adsorption of phages to their bacterial hosts and the mucus layer under certain environmental conditions (Fraser et al., 2007). We hypothesized that temperate phages may use the mucus barrier as a way of infecting incoming bacteria which may drive gene exchange and add another level of adaptation and evolution. It also may be a way of increasing genetic heterogeneity in a population of bacteria in late stage chronic lung infections that are traditionally thought to be somewhat clonal. We compare the inducible temperate phages of Pa found in the lungs of patients with CF and nCFBR using a metagenomic approach and study the complexity of putative functional traits the phage accrue through their continual adaptation and evolution in the chronic lower lung.

## Materials and methods

### Bacterial isolates and media

The Pa isolates in this study originate from clinical isolates collected at the Freeman Hospital and the Royal Victoria Infirmary; Newcastle Upon Tyne Hospital Trust, UK (10 pediatric CF isolates, 37 adult Cystic Fibrosis (CF) isolates, 17 < 10 year clinical diagnosis nCFBR isolates and 28 > 10 year nCFBR isolates). All bacterial cultures were propagated in Luria Broth (LB) media (Sigma Aldrich, Gillingham, UK), CaCl_2_ was added to Soft Agar [0.4% high clarity agar (Lab M Limited, Heywood, UK) and 0.01 M CaCl_2_ (Sigma Aldrich)] to promote phage adsorption; the cultures were incubated at 37°C for 18 h (+200 rpm if liquid culture). Full ethical approval has been given for this work (REC reference: 12/NE/0248).

### Prophage induction and re-infection

Lysogenic bacteriophages were chemically induced from bacterial isolates. In brief, overnight cultures were sub cultured 0.2% (v/v) (10 mL LB Broth, 0.01 M CaCl_2_). The phages were induced by stressing the bacterium with fluoroquinolone antibiotic, Norfloxacin (1 μg.mL^−1^) (Sigma Aldrich) for 1 h (37°C, 200 rpm). The culture containing norfloxacin was diluted (1:10) to limit the cytotoxic effect of the drug, so allowing for the cascade of phage induction to occur (Matsushiro et al., 1999). Phage lysates were filtered through a 0.22 μM filter (Scientific Laboratory Supplies, Hessle, UK) and stored at 4°C for <1 week. Phage lysates were also utilized to identify whether a phage from the lysate had the ability to re-infect the originating bacterial host. The lysates were spotted (10 μl) in dilution onto a lawn of originating host Pa cultured in 0.4% (w/v) agar + Luria Broth, overlaid on LB agar. Dilution identified plaques over possible pyocin production.

### Phage DNA isolation

Bacterial chromosomal DNA was attenuated using 1 μL of TURBO DNAse and 1 μL of RNAse Cocktail (Life Technologies Limited), prior to incubation at 37°C for 30 min followed with heat inactivation at 65°C and 0.5 M EDTA. NORGEN Phage DNA Isolation Kits (Geneflow Limited, Lichfield, UK) were used to purify viral DNA, in accordance with manufacturer's protocol. The NORGEN phage DNA isolation kit was chosen due to its optimal yield of phage DNA compared to the QIAGEN QIAmp MinElute Viral Spin Kit, Chelex extraction, and PEG8000 purification (Sambrook et al., 1989) whilst limiting bacterial chromosome contamination. A low level of bacterial chromosomal contamination was determined by PCR for the 16S rRNA gene but it was negated bioinformatically using the Khmer toolkit (Muyzer et al., 1993).

### Next generation DNA sequencing

The Illumina Nextera XT (Illumina, Saffron Waldon, UK) library preparation kit was used to prepare and multiplex the isolated phage DNA for next generation sequencing on the Ilumina MiSeq platform. A 2 × 250 cycle V2 kit was used for the loading and running of the sample. The DNA samples were diluted to 0.2 ng/μL (Qubit 2.0 DS HS DNA Kit [Life Technologies Limited]) prior to normalization and pooling. Paired end sequencing reads where provided as FASTQ files (NU-OMICS, Northumbria University at Newcastle, UK) and subject to downstream analysis.

## Bioinformatic tools

### Randomizing DNA reads using velvet V1.2.10

Velvet *de novo* genome assembler package shuffleseq.pl was used to randomly shuffle the FASTQ sequences to limit bias. The shuffled sequence output file was directly pipelined into the Khmer toolkit. Command line script can be found in Supplementary Material S6.

### Khmer toolkit

Khmer uses a probabilistic Bloom filter to separate out k-mer abundance and to group accordingly. This is achieved by creating hash tables that store specific k-mers and their counts using the default settings. The toolkit was utilized to remove very low-level bacterial contamination from the viral sequence data. The Khmer histogram clusters low abundance data and poor sequence data that would be linked to any residual bacterial chromosomal DNA where we can use the defining python script to select abundant viral k-mer sequencing data (Brown et al., 2012). Command line script can be found in Supplementary Material S6. For each individual sequence file we assessed the out.hist files graphed in excel and manually remove the error k-mer peak. These output files then can be pipelined to MG-RAST once the data is renamed using “Rename Sequences” v 0.0.11 (Blankenberg et al., 2010).

### Renaming FASTA files

Following the separation of the raw data into separate sequence files via Khmer, the files were converted from FASTQ to FASTA using a python script (Khmer package). Sequences were renamed numerically using “Rename Sequences” v 0.0.11 (Blankenberg et al., 2010). These 92 viromes where then uploaded to MG-RAST. From the 92 files and sequence cleanup 10 files had under the threshold of data allowed for uploading onto MG-RAST. However, we still assembled each of these samples using the three assemblers and used them to search for any putative Ig-like domains.

### MG RAST

The KEGG generator function was used in order to show possible differences in the biochemical pathways between the phage isolated from the 2 clinical origins and stratified in the methods section. The maps were generated with the MG-RAST default setting: 60% sequence similarity of 15 amino acids. The hierarchical classification system tab was used in order to generate Principle Component Analysis (PCoA) on the samples relating to their functionality, the data was normalized and drawn according to the Minkowski distance with MG-RAST default settings.

### Three-way genome assembly comparison

Before we could search the sequence data for putative carbohydrate binding motifs using GeneWise2 we perform a three-way assembly study using SPAdes v 3.1.0, Velvet optimizer v 2.2.5 and IDBA-UD v 1.1.1. An overview of the assemblies is provided in Supplementary S5 that details each assembly comparison showing N50 scores, number of contiguous sequences derived and the largest contig size. **Figure 5A** shows the ability to detect Ig-like domains compared between the assemblers.

### HMM/PFAM database searches using genewise2 V2.2.0

GeneWise2 was used to search a database of 92 sequence files against the Pfam database of 40 amino acid based HMMs as shown in Table 1. HMMER v 3 was used to revert the HMMs from version 3 to version 2, so GeneWise2 could recognize these files. GeneWise2 algorithms 6:23 and 21:93 were used and comparisons drawn with Jalview v 2 (Waterhouse et al., 2009). The gene locations of the resulting positive results were compared to the putative ORF associated with the GeneWise2 identification.

**Table 1:** **The 40 Pfam databases used in GeneWise2 and adapted from Fraser et al. (2006)**.

**SCOP superfamily**	**PFAM name**	**Accession number**
Ig	V-set	PF07686
	I-set	PF07679
	C2-set	PF05790
	C1-set	PF07654
	Ig	PF00047
	Ig_2	PF13895
	ICAM_N	PF03921
E-SET	Alpha_amylase_N	PF02903
	arrestin_N	PF00339
	arrestin_C	PF02752
	CelD_N	PF02927
	peptidaseC25	PF03785
	TIG	PF01833
	RHD	PF00554
	DUF291	PF03442
	Filamin	PF00630
	He_Pig	PF05345
Fibronectin type 3	FN3	PF00041
	tissue_fac	PF01108
	lep_receptor_Ig	PF06328
PKD	PKD	PF00801
	PPC	PF04151
	HYR	PF02494
β-Galactosidase/β-Glucuronidase	Glycol_hydro_2	PF00703
Cu, Zn Superoxide dismutase-like	Sod_Cu	PF00080
PapD-like	Pili_assembly_C	PF02753
	pili_assembly_N	PF00345
Invasin/intimin cell-adhesion fragments	Big_1	PF02369
	Big_2	PF02368
	Big_3	PF07523
	Big_4	PF07532
Clathirin adaptor appendage domain	Alpha_adaptin_C2	PF02883
Transglutaminase N-terminal domain	Transglut_N	PF00868
Cadherin-Like	Cadherin domain	PF00028
Actinoxanthin-like	Neocarzinostatin family	PF00960
CBD9-like	Domain of unknown function	PF06452
laminA/C globular tail domain	Intermediate filament tail domain	PF00932
Other Ig-like	C type Lectin	PF00059
	BACON	PF13004
	MucBP	PF06458

They were individually selected as they encode putative carbohydrate binding domains.

## Results

Putative mixed viral communities were induced from a cross-sectional panel of 92 *Pseudomonas aeruginosa* (Pa) bacterial isolates, 47 isolated from CF patients and 45 nCFBR stratified further by patient clinical information detailed in the methods. Phage DNA was isolated away from bacterial chromosome and any low level remaining bacterial DNA was removed bioinformatically using Khmer toolkit as described both in the methods and Supplementary Material.

### MG-RAST derived KEGG pathway analysis

Eighty two from ninety two isolates were analyzed through MG-RAST and KEGG analysis due to 10 of the sequencing files having insufficient sequence data for analysis. The pathways that possibly confer putative function are shown in the Supplementary Data alongside the raw data generated from each KEGG pathway (S1 and S2 respectively). Using KEGG pathway analysis through MG-RAST we defined the “incidence” of DNA sequences with shared similarity with a known metabolic pathway stored in the KEGG database. An overview of the number of reads per sample is shown in Supplementary S3 whilst the KEGG derived EC values downloaded from the KEGG generator tab on MG RAST are shown in Supplementary Table S4.

**Figure 1:**
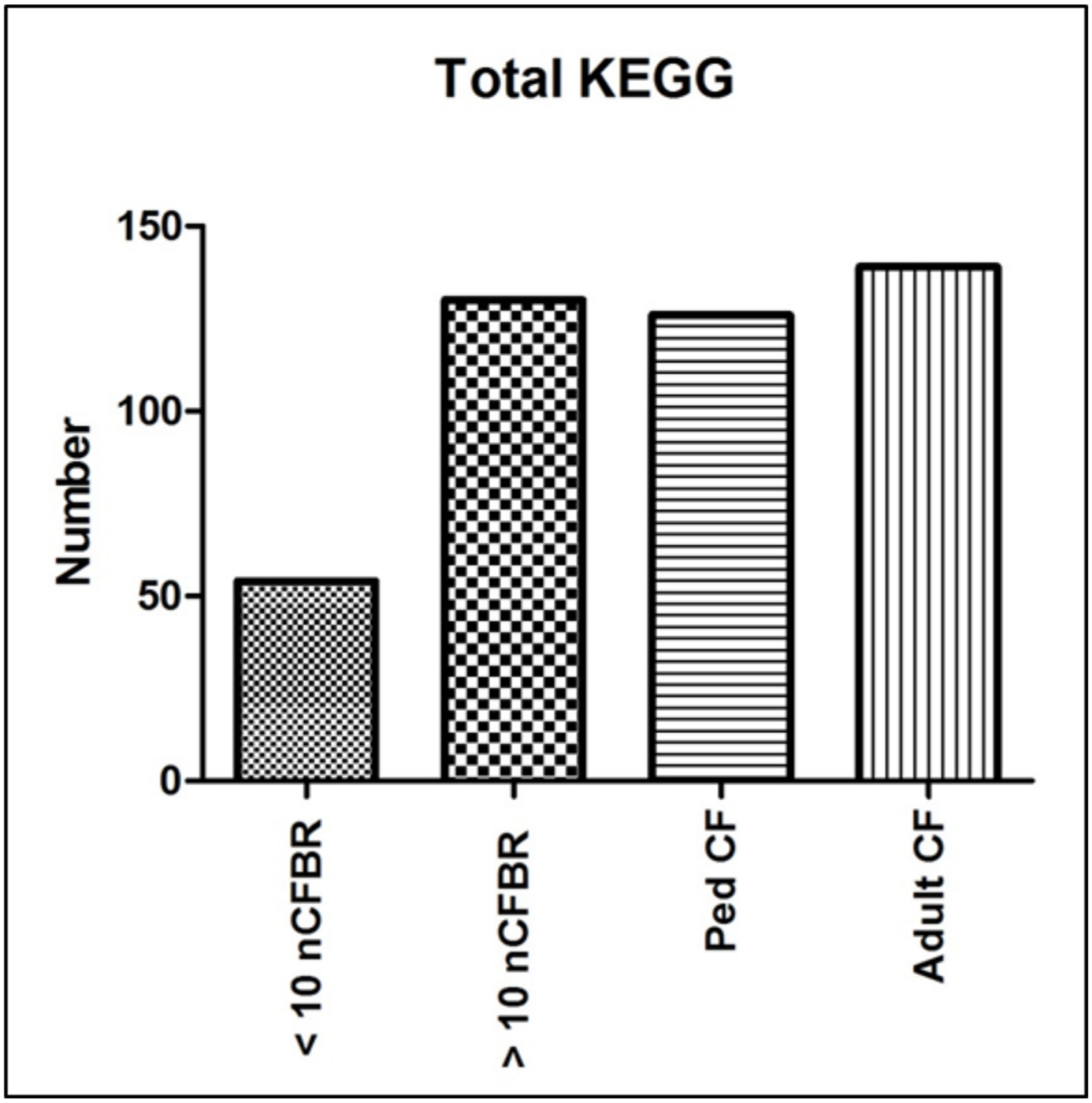
**Total KEGG putative function identifications derived for phage lysates**. Each putative functional pathway is represented as a single identification regardless of the amount of times that the putative function may have been conferred in each of the clinical subgroups. The highest rates of identification are seen in the lysates that originate from adult CF patients whilst the lowest rates of identification are seen in the lysates from <10 year clinical diagnosis nCFBR patients.

**Figure 2:**
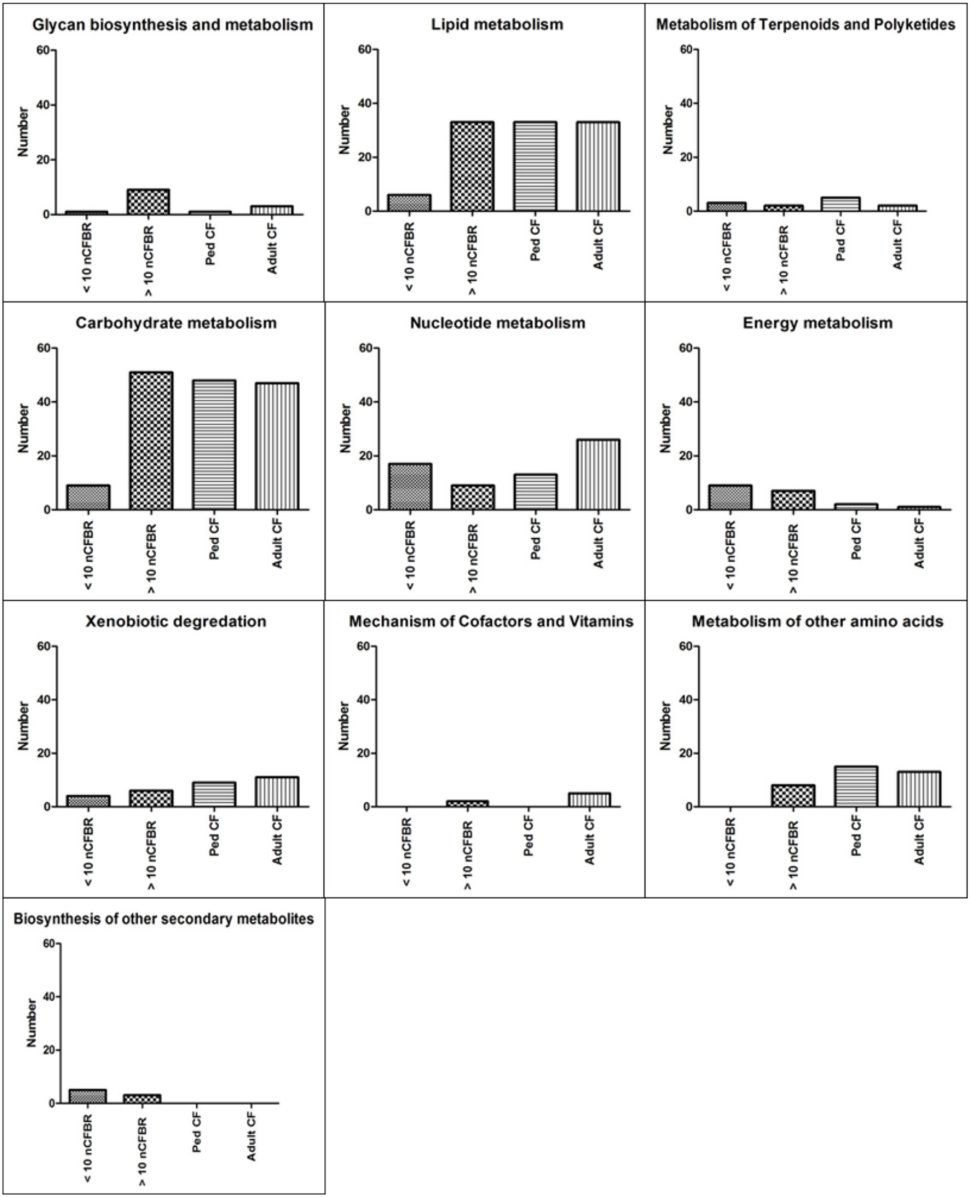
**Incidence of amino acid similarity to KEGG functional pathways generated by MG-RAST**. These data are further stratified sub-etiology to; pediatric CF (ped CF), adult CF, < 10 year clinical diagnosis nCFBR and >10 year clinical diagnosis nCFBR. Each putative functional pathway is represented as a single identification regardless of the amount of times that the putative function may have been conferred in each of the clinical subgroups. These KEGG pathways can be used to confer possible functionality and difference between the disease states. A clear increase in the number of KEGG pathway identifications is linked to patient age regardless of disease etiology (“Glycan biosynthesis and metabolism,” “Xenobiotic degradation,” and “Mechanism of Cofactors and Vitamins”). This pattern is reversed in some panels with a reduction in the number of identifications decreasing as the disease progresses regardless of etiology (“Metabolism of Terpenoids and Polyketides” and “Energy Metabolism”). When looking Nucleotide metabolism there is a decrease in the amount of identifications between <10 year clinical diagnosis nCFBR and >10 year clinical diagnosis nCFBR but an increase between ped CF and adult CF patients. In two panels (“Lipid Metabolism” and “Metabolism of other amino acids”) there is also an increase in the number of identifications seen as nCFBR patients disease state progresses but a decrease in the number of hits as a CF lung deteriorates. When looking at the final panel (“Biosynthesis of other secondary metabolites”) no similarities are detected for the CF and nCFBR patients regardless of progression of disease.

Using KEGG pathway analysis Figure 1 identifies the presence of different metabolic pathways that are stored in this database. We stratify this further using the clinical information detailed in the methods. We identify an increase in KEGG derived pathways that link to the duration of clinical disease in adult CF and >10 years nCFBR. However, nCFBR (<10 year clinical diagnosis) lysates have the lowest number of KEGG pathway incidences. There is an overall increase in the number of identifications as both diseases progress. This may show phage adaptation and accrual of genes that possibly aid the fitness of the bacterial host within this environment. Figure 2 shows bar graphs demonstrating the incidence of KEGG identifications that have been stratified both by clinical etiology but also by the defining zones of the KEGG atlas. The KEGG pathway analysis identifies gene regions and links to functional pathways relating to metabolism and signaling. Increases in functionality that can be linked to disease progression include; glycan biosynthesis and metabolism; xenobiotic degradation; mechanisms of cofactors and vitamins. An overall decrease in identifications was identified in pathways relating to; metabolism of terpenoids and polyketides, and energy metabolism. However, when looking at some areas of the KEGG atlas there are differences between the CF and nCFBR lysates. Lipid, carbohydrate and amino acid metabolism increases as nCFBR progresses where the opposite is apparent for CF patients. The reverse of this trend is observed in hits relating to nucleotide metabolism, with a decrease in hits in nCFBR patients compared to an increase in CF patients. It is noteworthy that when comparing identifications relating to the biosynthesis of secondary metabolites it is clear that there are no hits in a CF phages background.

#### MG-RAST derived PCoA

**Figure 3:**
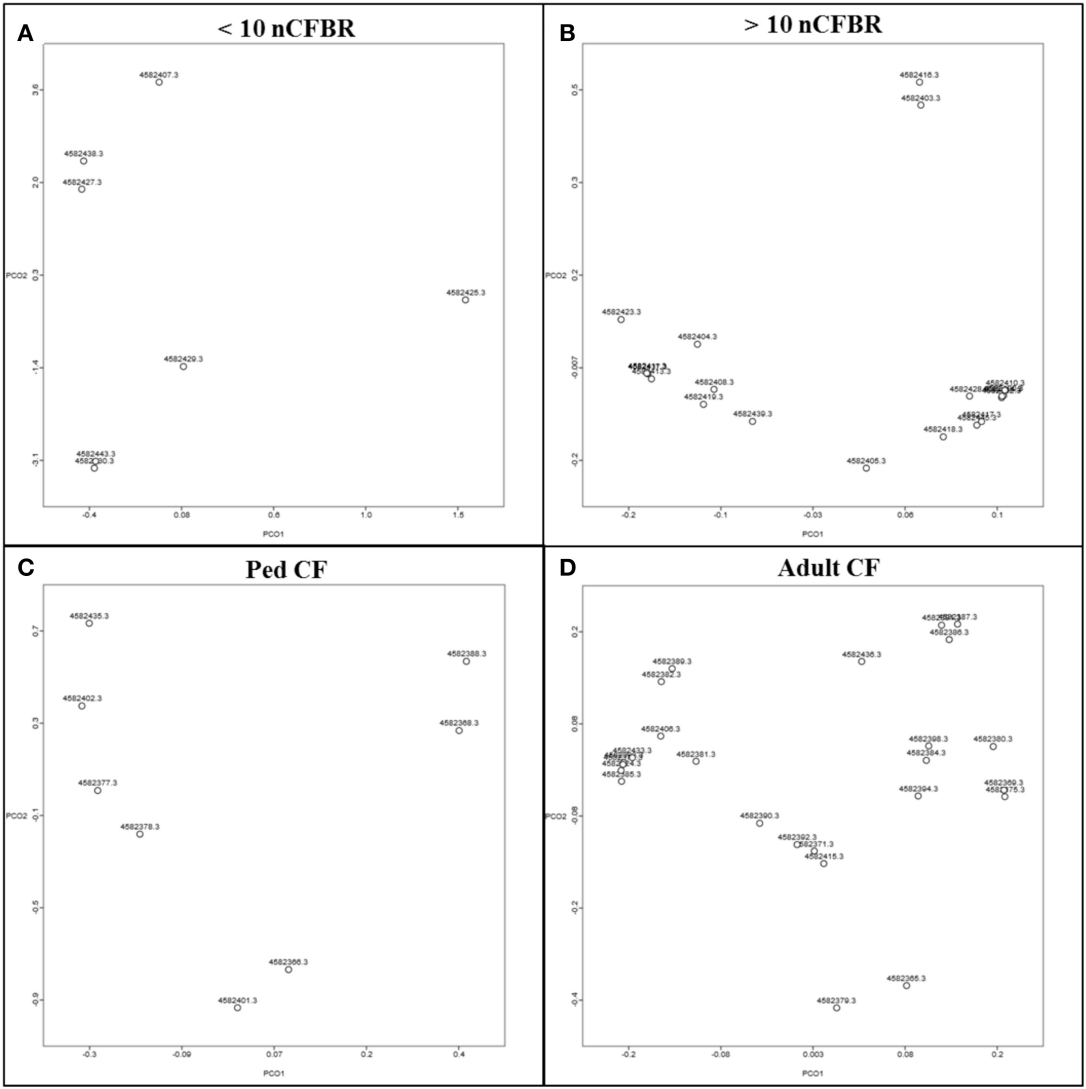
**Principle component analysis drawn in MG-RAST according to COG and the Minkowski distance**. They indicate that the phage lysates postKhmer analysis have no relatedness to the presence of a Big_2 domain in each sample. **(A)** Represents <10 year clinical diagnosis nCFBR samples, **(B)** represents >10 year clinical diagnosis nCFBR samples, Ped CF samples are shown in **(C)** and the adult CF samples are shown in **(D)**.

Principal component analysis (PCoA) generated by pan differences in functionality (Figure 3) illustrates that the phage samples show unrelated functionality and that there is no observable link between the phage functionality on the PCoA. This suggests that even though differences in function are seen when looking at the KEGG atlas (Figures 1, 2); these differences are not discrete enough to lead to separation on the PCoA plot and thus show that not all phage carry all traits.

#### Phages ability to Superinfect Pa

**Figure 4:**
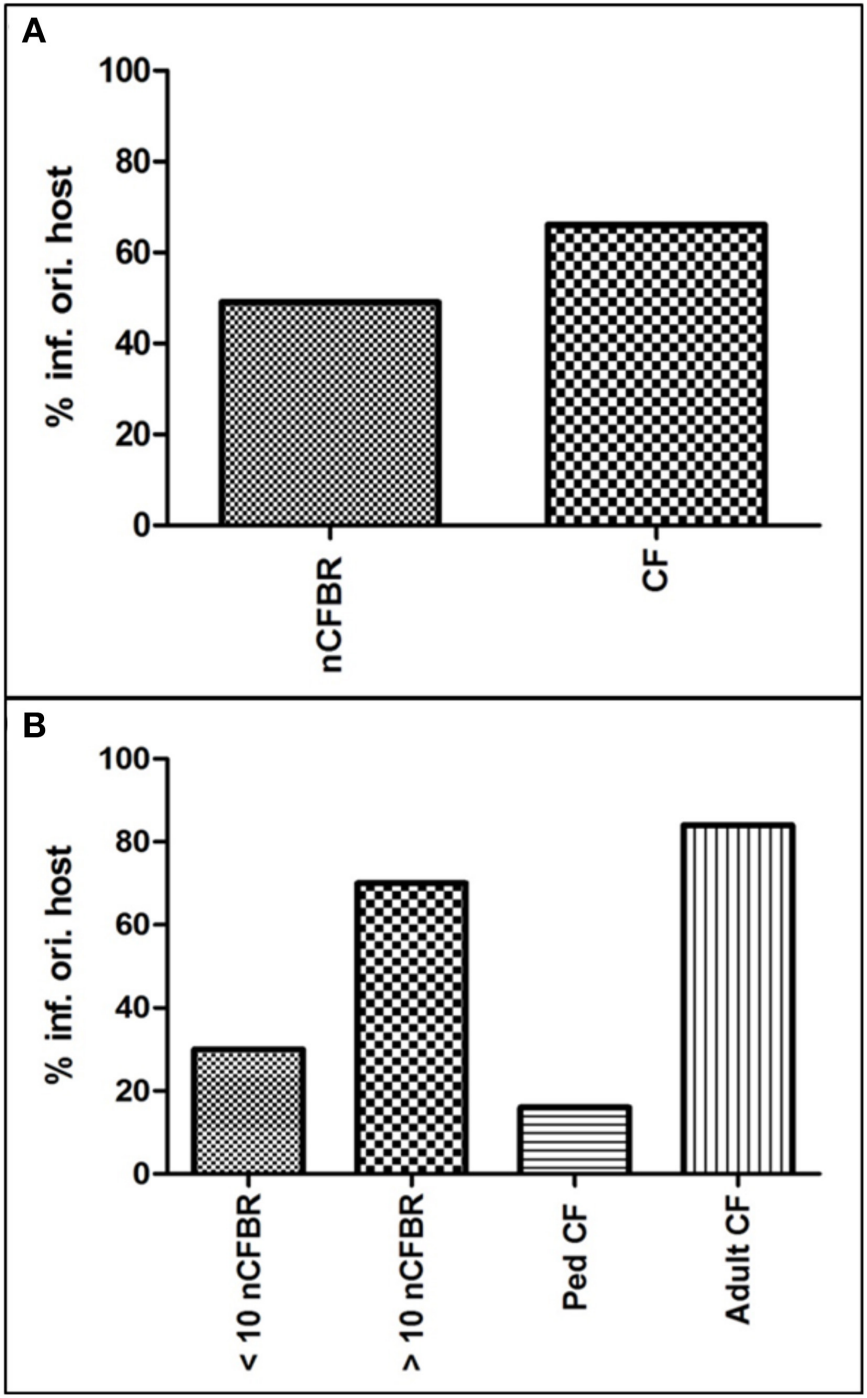
**Graphical representation of the percentage of phages that are capable of re infecting their originating host (% inf. ori. host = % infection of originating host). (A)** Shows the percentage of phage lysates from both nCFBR and CF, with CF phage having greater ability to infect their originating host. **(B)** shows the stratification of these rates shown in **(A)**, illustrating that this trait is acquired over time in phages isolated from both clinical etiologies.

A large number of phages that were chemically induced in a previous study from the 94 isolates have the capability of re-infecting their originating bacterial host (Figure 4). Stratification according to disease origin has been shown in Figure 4A where 49% of nCFBR related phages had the ability to re-infect the originating host compared to 66% of the CF Pa induced phages. In Figure 4B further stratification illustrates that 16% of pediatric CF Pa phages within these samples and 84% of the adult samples have the ability to re-infect. This trend is also seen in nCFBR patients with 30% <10 years clinical diagnosis and 70% >10 years clinical diagnosis able to re-infect their originating host.

#### Presence of Ig-like domain Big_2

**Figure 5:**
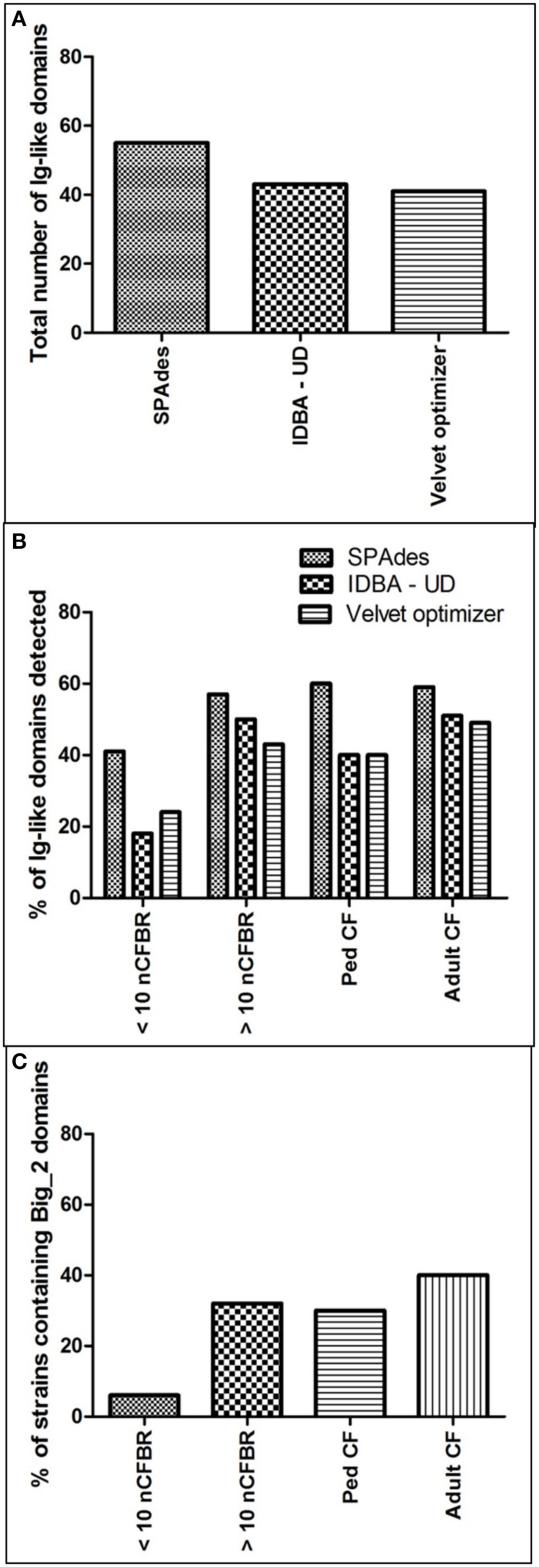
**(A)** Describes the total number of Ig-like domains identified using Genewise2 subsequent to assembly by SPAdes, IBDA-UD, and Velvet Optimizer. **(B)** Offers the percentage of the total number of Pa isolates from the 4 clinical groups with one or more Ig-like domain. **(C)** Focuses on the percentage incidence of the Big_2 domains which attracted the highest bit scores of detection using GeneWise2.

The frequency of Ig-like binding domains is described in Figure 5. It illustrates that the frequency of Big_2 domains increases alongside the longevity of disease. This has been calculated as a percentage of the total number of phages for each of the clinical strata, which have been seen to contain a Big_2 domain (CF: pediatric CF phages 30%, adult CF phages 40%; nCFBR: <10 years clinical diagnosis 6%, >10 years clinical diagnosis 32%). This work is unique as these domains have not been observed on phage genomes to this level previously, especially on phages with known clinical origins.

#### Identification of Ig-like binding domains

**Figure 6:**
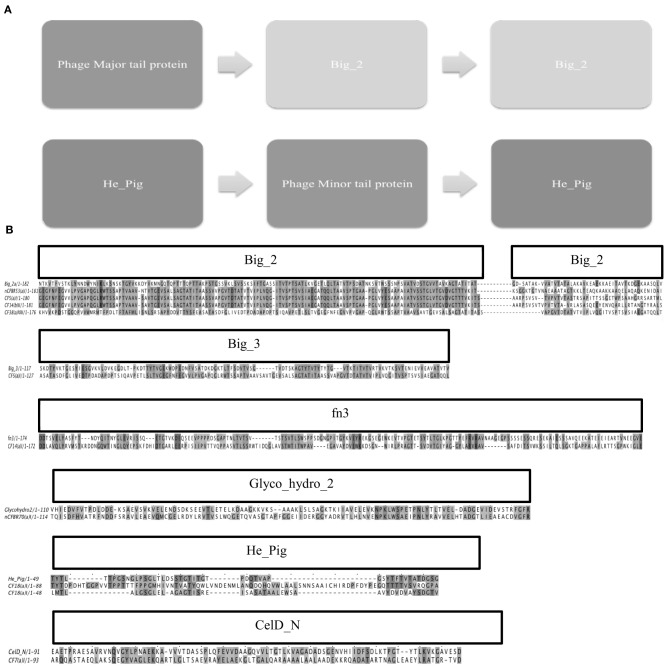
**(A)** Shows the domain architecture of the double Big_2 domain that was identified in a subset of the phage lysates from the clinical samples, the second image is the proposed domain architecture for He_Pig. The He_Pig domains however, span over different genes adjacent to the Phage minor tail protein. **(B)** Shows the sequence alignments of the Ig-like domains; Big_2, Big_3, Fn3, Glyco_hydro_2, He_Pig and CelD_N found in phage proteins. The sequence alignment of Big_2 is represented along with 4 variations that were seen in this study; all the samples have a high similarity to the Big_2 domain represented on the top line. Big_3 and Fn3 both have a lower sequence identity to the known sequence but this alignment was conserved between all the phages that were identified to contain these domains. The Glyco_hydro_2 domain was seen in only one phage sample (> 10 clinical diagnosis nCFBR isolate). The He_Pig double domain was seen in 42 of the phage samples, 70% of which have the amino acid alignment shown above.

Big_2 domains were only identified with both GeneWise2 6:23 and 21:93 algorithm and these were seen in 28 of the 92 mixed phage samples. All bit similarity scores were above the recommended cut-off of 25, were considered significant for gene prediction (Fraser et al., 2006). The domain architecture found in these samples is shown in Figure 6A. It was seen that the Big_2 domains were found in duplicate and associated with the major tail protein. It was also seen using GeneWise2 algorithm 21:93, where a double He_Pig domain presented in a large subset of the Pa phages as a double motif in putative structural genes flanking a putative minor phage tail protein. The He_Pig double domain was seen in 42 of the phage samples, over 70% of which have the amino acid alignment shown in Figure 6B. All the other hits to the He_Pig domain showed poor bit scores and were thus not aligned in Figure 6B.

When the GeneWise2 21:93 algorithm was used, it was seen that a Big_3 domain was present overlapping (by 89 amino acids) the first Big_2 domain which may link to a frameshift that has been described in other Ig-like binding motif architecture (Fraser et al., 2006). This Big_2, Big_3 combination trend was seen in all of the samples, except for four where the algorithm failed to report any significant similarity. We also identified another domain in 10 of the samples called Fn3. The domain alignments for Big_3, Fn3, Big_2, glycol_hydro_2, CelD_N, and He_Pig are shown in Figure 6B. The Glyco_hydro_2 domain was identified but not associated with a putative structural protein. Upon utilizing a BLASTn search the Fn3 domain was located in a phage tail assembly protein. Similarly the ORF for Big_2 was also identified in a structural major tail protein gene, this time the major tail 2 protein. Other Ig-like domains have been identified but they all overlapped the first Big_2 domain including; PPC, Peptidase_C25_C, Big_3, and Big_4 all had too low bit scores.

## Discussion

This study reports the first use of metagenomic approaches to identify the inducible temperate bacteriophages isolated from single clonal cultures of *Pseudomonas aeruginosa* (Pa) colonizing the lungs of patients with CF and nCFBR. These data were further compared to the clinical information provided for each patient sub-group. A noteworthy observation is the evolution in complexity of the viral population linking to possible functions that aid bacterial fitness and therefore, viral sustainability in chronic lung infections. Key findings are the high frequency of Ig–like motifs found in the temperate phages of Pa with known clinical origin and also the increase in complexity of putative phage function when adapting to the progressive disease state of the lower lung.

When analyzing the metagenomic results generated via KEGG analysis through MG-RAST, it is clear to see an increase in complexity of function and possibly the level of adaptation occurring between the phage in accordance with either patient age or colonization time with Pa (Figures 1, 2). Using KEGG we also observed specific functional trends encoded by temperate phages that are both similar and disparate between the disease states. An increase is seen in the number of identifications for glycan biosynthesis and metabolism; this is possibly due to these functions being associated with cell wall synthesis in the bacteria and possibly associated with inflammation in the lung. The CF lung also contains a large amount of human produced mucins which are covered in glycans, so it is possible that the phage are transporting functions relating to glycan biosynthesis in order to increase the degradation of these mucin's and thus promote bacterial growth in the CF lung. Phages therefore, may utilize these functions in order to survive, by driving further inflammation in the lower lung environment offering preferential selection for the bacterium. When focusing on certain subsections such as “carbohydrate metabolism” and “nucleotide metabolism,” apparent differences are seen between some of the disease sub-groups and these may be caused by alteration in metabolic precursors within the lung environment where addition by the phage offers an alternate pathway of metabolism. It is surprising to see the low level of association with “energy metabolism” in adult CF phage metagenomes and in >10 year clinical diagnosis nCFBR phage, but it may be possible that this is due to colonizing bacteria, in later stages of disease progression, may have evolved the perfect metabolism to survive and thus this relates to gene subtraction over addition. This also may be linked to the propensity of these Pa isolates to sustain in a biofilm.

The increase in the ability of certain phage communities to encode functionality that enables bacteria to degrade xenobiotic compounds is illustrated to evolve alongside the bacterium. It is notable that this also links to the previous reports of increasing antibiotic resistance in Pa isolated from the chronic lung (Winstanley et al., 2009). This pattern of increasing incidence of putative function also correlates to the increasing timeline of these diseases. This is illustrated by increases in incidence of metabolic pathways of cofactors and vitamins that have a role in generating biological activity. It may be possible that these cofactors are being encoded by the phage to aid bacterial survival and fitness. It must also be taken into account the apparent lack of these factors early on in these diseases may show a possible evolutionary timeline. The sizeable work undertaken here shows that adaptation of phage communities is apparent, with the amount of bacterium and possible number of phages offering confidence. This notable observation emphasizes the complexity and detailed nature of chronic lung infections and how phage evolution over time may affect the phenotype of Pa which in turn will have an overall impact on disease progression.

Bacteriophages evolve strategies alongside their bacterial host that promote infection, propagation and offer fitness to their host range. The inflamed lung is rich in mucus and the Pa isolated are variable in phenotype with some Pa expressing a mucoid surface. Therefore, it was pertinent to compare the BAM model of lytic phages as it may offer a way that temperate phages infect and transduce across their host range in the lower lung. Initially in this investigation we focused our attention on specific carbohydrate binding domains including Bacteriodetes Associated Carbohydrate Often N–terminal (BACON) (Mello et al., 2010) as this has been shown to be involved in the BAM model (Dutilh et al., 2014). However, searching Pa phage metagenomes using GeneWise2 identified no BACON domains in the panel of 92 phage samples. This does not refute the BAM model in this setting as the phage may bind to the Pa bacterial host via another carbohydrate or glycoprotein. Out of the 92 isolates and their associated phage, a Big_2 domain was observed in at least one of the phage's isolated from various clinical backgrounds (3 pediatric CF isolates, 15 adult CF isolates, 1 < 10 year clinical diagnosis nCFBR isolates and 9 > 10 year clinical diagnosis nCFBR isolates). When looking at the presence of the He_Pig motif it was identified that its occurrence was equal in both etiologies with a higher propensity in adult CF phage and >10 year Pa colonization nCFBR phage (17 and 14 respectively), putatively showing a role in phage adaptation. There were hits for the He_Pig domains in both pediatric CF phage (4) and <10 year clinical diagnosis nCFBR phage (7). Importantly we use three bioinformatics packages to assemble the DNA of each phage metagenome. We report that SPAdes and IBDA-UD are fairly comparable in performance, although we identified higher numbers of Big_2 domains using SPAdes. In addition Velvet optimizer yielded the poorest assembly when specifically targeting Ig-like domains with GeneWise2. Importantly though the Velvet derived assembly contained an Ig-domain (PF00047) that both SPAdes and IBDA-UD failed to assemble. We would therefore recommend utilizing all 3 assemblers for future studies in this developing field.

Ig-like domains have been identified on approximately 25% of all sequenced *Caudovirales* genomes, there are three distinct families which are only identified in *Caudovirales* phage (Big_2, I-Set, and fn3) (Fraser et al., 2007). These domains on *Siphoviridae* and *Podoviridae* are located on Major Head, Major Tail and Tail Fiber proteins whilst in *Myoviridae* they are located on HOC, Fibritin and Baseplate proteins (Fraser et al., 2007). The lytic T4 phage in the BAM model shows the phage associating with mucus via the head HOC protein, this is seen with Electron Microscopy (EM) as the domain protrudes from the surface of the phage (Crusoe et al., 2010). Ig–like domains have been identified in the tail tube protein of *E. coli* bacteriophage λ, the exact reason for these Ig-like domains in λ is not fully known but when these domains are truncated, the phage has been seen to become more temperature sensitive (Katsura, 1981; Pell et al., 2010). These Ig–like domains may have accessory roles in bacterial infection rather than essential roles but their ubiquitous nature does potentially indicate there is an evolutionary advantage for the phage containing these domains (Pell et al., 2010; Barr et al., 2013). We show that Pa phages assembled in this study have a double Big_2 domain. The He_Pig domain was also seen in a double motif which flanked a putative minor phage tail protein (Figure 6). He_Pig is an Ig-like domain that is found in hemagglutinin and cell surface proteins, so it is possible that this domain is involved in the BAM model. The major sequence variations seen between the 4 types of the double Big_2 motif were found in the second Big_2 domain, Figure 6B. All these domains had a bit score above the proposed score of 25 and had a gap ranging from 18 to 6878 bp.

The BAM model reports on the role of the capsid-displayed, Ig-like protein domain of phage T4, and suggests a mechanism for its adherence to mucus. The putative carbohydrate-binding domains identified in this study appear to be associated with phage tail protein structures, rather than capsid domains. The presence of Ig-like domains therefore, on the phage isolates may indicate that the BAM model is functional for temperate phage but as a mode of infection and propagation rather than phage mediated immunity. We determine that rapid evolution may be making the overall diversity of phage Ig-like domains very large and so this may hinder the detection of these domains with GeneWise2 (Fraser et al., 2006) hence our suggestion of utilizing multiple assembly software. When comparing the clinical data associated with the bacterial isolates there is no correlation between the detection of Ig-like domains and the severity of disease.

**Figure 7:**
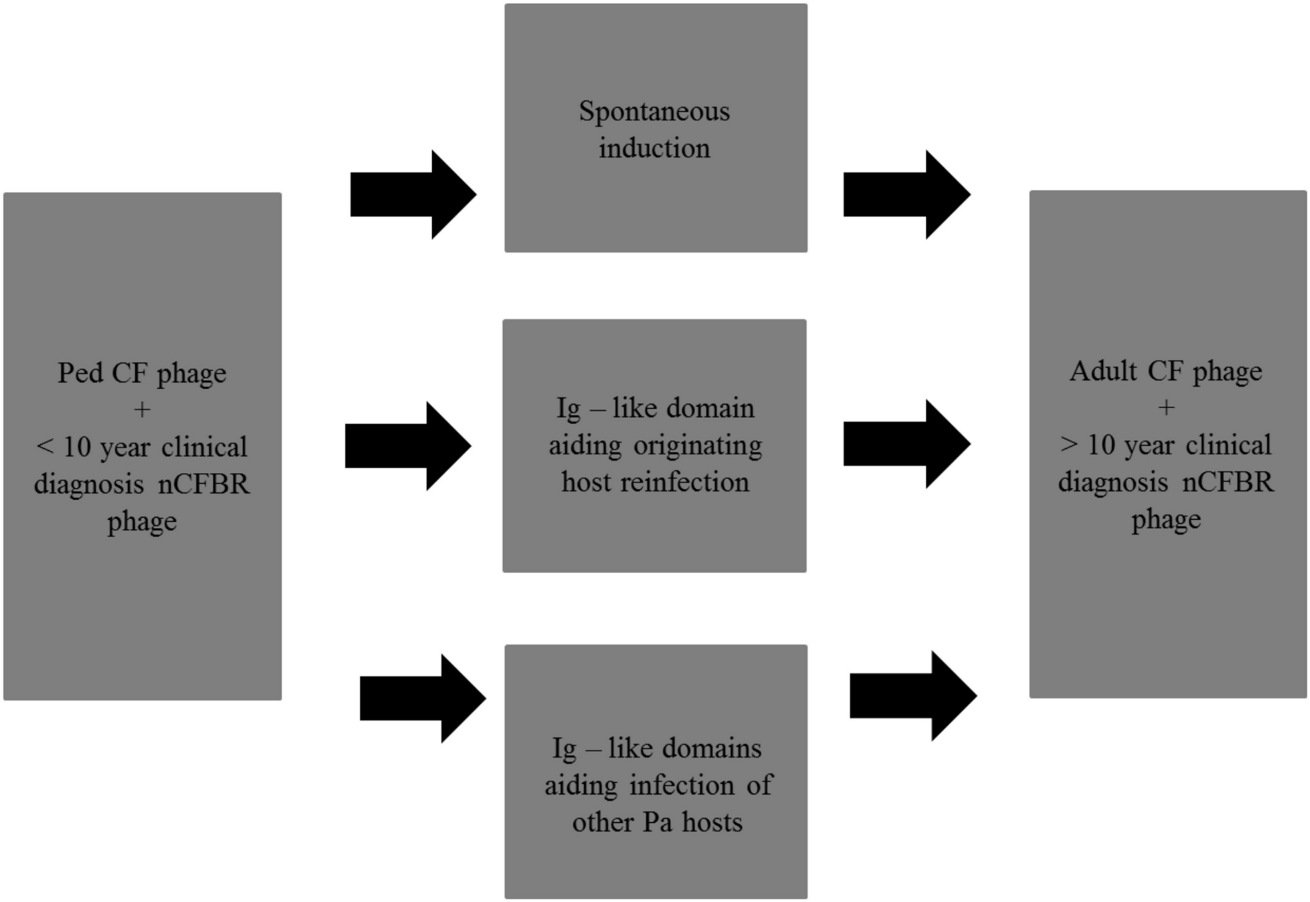
**Possible strategies that Pa phage can adapt over time in the chronic lung, various proposed mechanisms are included**. Ped refers to pediatric CF patients in this figure and in future figures.

This metagenomic project has indicated that the phage isolated from Pa derived from CF and nCFBR patients can associate with carbohydrates either on the bacterial host cells or in the mucus via Ig–like domains. When looking at the samples containing a Big_2 domain and/or a He_Pig domain, it was seen that they were more common in adult CF phage and >10 year clinical diagnosis nCFBR phage, possibly indicating that the presence of these domains has an evolutionary advantage for the phage's longevity in the chronic lung. This is the first time that this level of frequency has been observed for Ig–like domains in clinical isolates. There are multiple pathways which the Pa phage may utilize in order to adapt and offer fitness to its Pa host in the chronic lung environment (Figure 7). Through these data we can propose possible strategies the phages have adopted in order to sustain the ability to infect and propagate within the lower lung, alongside etiological and evolutionary difference in the bacterial host. We have previously determined that temperate phages of Pa have high levels of spontaneous induction, with elevated levels being observed when Pa is in the early rather than mid-exponential growth phase. We hypothesize that phage are released from their host cell early to avoid becoming encased in a potential biofilm and have vegetative cells to infect. However, some of the phage that contains the Ig–like domains may aid adsorption to the cell from which they originate, or aid attachment and infection of other cells in their direct environment. Addition of traits such as adherence to the mucus lining the lower airways in order to increase their longevity in the chronic lung environment is also pertinent as an adaptive strategy.

This project has used metagenomics to show the array of adaptive mechanisms phage communities accrue over time in the lung environment. This work highlights the novel nature of metagenomics to understand complex communities without the need for a sensitive bacterial host or the necessity to enrich bacteriophage numbers and culture independence. We look at the “total arsenal” of a mixed phage community being induced from a clonal population and look at their possible impact on their next host in terms of infection and subversion. We map the effect that these mixed phage communities have on the functionality of their host or the strategies including BAM that have evolved over time. Using single phage lysates would not represent the complexity of the phage communities from each bacterium and their infection strategies in the chronic lung environment.

In conclusion, this study characterizes the inducible temperate phages found in Pa isolates sampled from the lungs of patients with CF and nCFBR. We provide the first evidence of how the complexities of the phage genomes possibly adapt over time and acquire accessory genes linking to key metabolic and signaling processes that may aid phage survival and bacterial fitness within the chronic lung. This study reports for the first time, to the best of our knowledge, the identification of multiple Ig-like domains on Pa temperate phages structural genes with such high frequency. There are increasing concerns over progressive anti-microbial resistance and the slow development pipeline for new antibiotics, so there is a real need for a developing novel paradigms and methods to overcome or further understand these challenges. These data offer temperate phages as a possible target in the resistance/ persistence pathways. Furthermore, they raise an additional clinical concern - could phage cross infection be a new challenge?

### Conflict of interest statement

The authors declare that the research was conducted in the absence of any commercial or financial relationships that could be construed as a potential conflict of interest.

## Footnotes

^1^WHO. Available online at: http://www.who.int/genomics/public/geneticdiseases/en/index2.html#CF [Accessed February 20, 2014].
