# Supplementary material for: A metagenomic approach to characterize temperate bacteriophage populations from Cystic Fibrosis and non-Cystic Fibrosis bronchiectasis patients
Source: Front Microbiol. 2015 Feb 18;6:97. doi: 10.3389/fmicb.2015.00097 (PMC4332376; doi:10.3389/fmicb.2015.00097)
Supplement: Supplementary file 1 [file DataSheet1.DOCX]

**Supplementary data:**

**
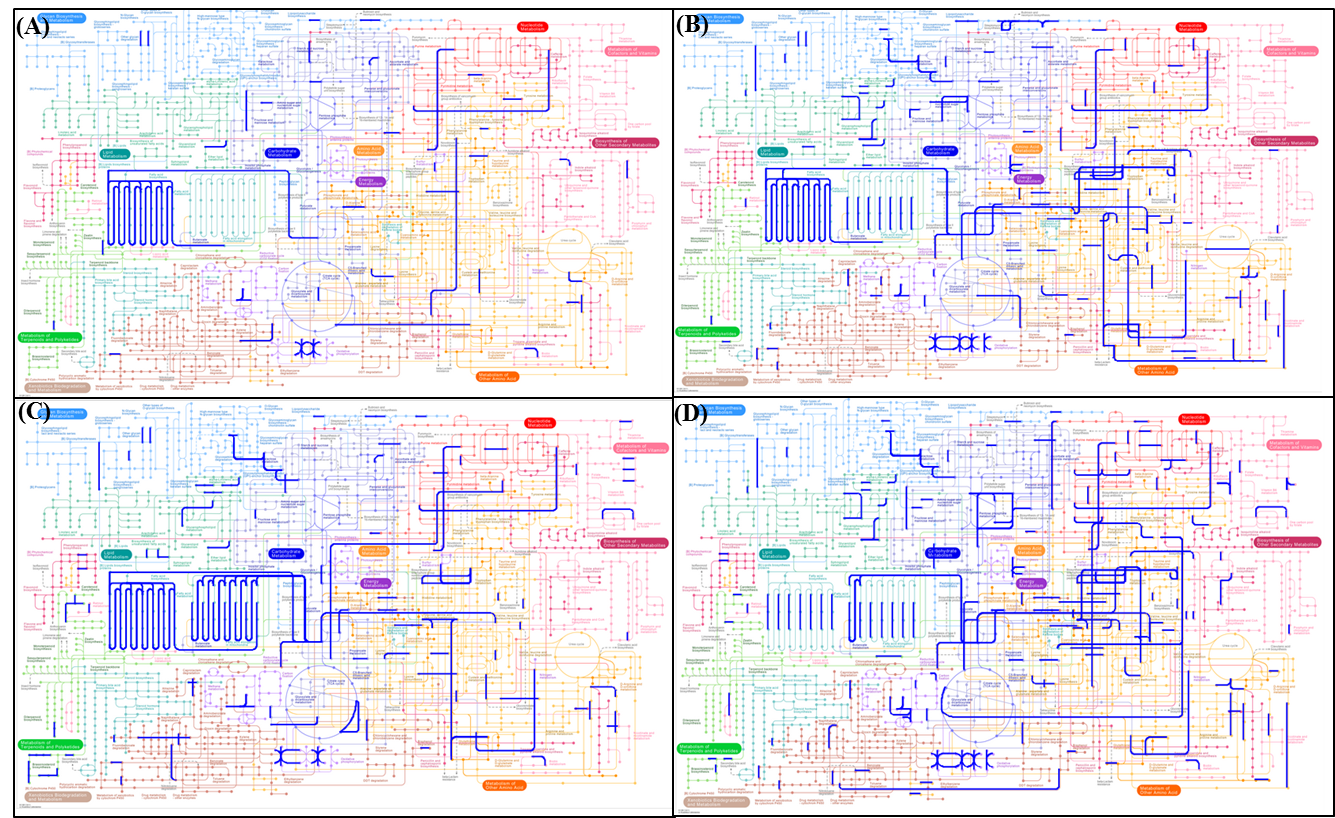
**

**S1 -** KEGG map generated with MG RAST post Khmer processing. Panel A represents the < 10 year clinical diagnosis nCFBR phage, panel B represents the > 10 year clinical diagnosis nCFBR phage. Panel C represents ped CF isolates and adult CF isolates are shown in panel D.

**S2 –** Break down of all the components of the KEGG images (post Khmer) with the potential functionality hits being split according to subsystems as well as clinical etiology.

| **GLYCAN BIOSYNTHESIS AND METABOLISM** | **KEGG Identification** | **Putative function** |
| --- | --- | --- |
| **Pediatric CF** | Lipopolysaccharide biosynthesis | Cell wall function |
| **< 10 year clinical diagnosis nCFBR** | Glycosphingolipid biosynthesis | Involved in inflammation in chronic lung diseases |
| **Adult CF** | Other types of O-glycan biosynthesis | Mucin binding domain – phage may encode to aid its attachment (serine/threonine base) |
|  | Glycosphingolipid biosynthesis - ganglio series | Involved in inflammation in CF |
|  | Lipopolysaccharide biosynthesis | Synthesis of lipopolysaccharides |
| **> 10 year clinical diagnosis nCFBR** | Sphingolipid metabolism | Involved in inflammation in chronic respiratory diseases |
|  | Glycosphingolipid biosynthesis - lacto and neolacto series | Involved in inflammation in chronic respiratory diseases |
|  | Other types of O-glycan biosynthesis | Mucin binding domain – phage may encode to aid its attachment (serine/threonine base) |
|  | Various types of N-glycan biosynthesis | Involved in correct protein folding (aspartate base) |
|  | Galactose metabolism | Energy – sugar |
|  | Other glycan degradation | Degradation of linked monosaccharides |
|  | Glycosaminoglycan degradation | Long unbranched polysaccharides made of repeating disaccharide units |
|  | Glycosphingolipid biosynthesis - ganglio series | Involved in inflammation in chronic respiratory diseases |
|  | Lysosome | Break down of biomolecules including proteins, amino acid, lipids and CHO – found in animal cells |
| **LIPID METABOLISM** | **KEGG Identification** | **Putative function** |
| **Pediatric CF** | Sphingolipid metabolism | Involved in inflammation in CF – possible drug target |
|  | Other types of O-glycan biosynthesis | Mucin binding domain – phage may encode to aid its attachment |
|  | Glycosphingolipid biosynthesis - ganglio series | Involved in inflammation in CF |
|  | Fatty acid degradation | Degradation of fatty acids |
|  | alpha-Linolenic acid metabolism | Alter the Fatty Acids in CF – have an effect on potential CF diet management |
|  | Fatty acid metabolism | Fatty Acid metabolism |
|  | Valine, leucine and isoleucine degradation | These are previously seen to be elevated in the CF lung |
|  | Geraniol degradation | Geraniol (bacterial nutrient source) |
|  | Benzoate degradation | Utilize alternative Carbon sources – longevity |
|  | Ethylbenzene degradation | Plant originating metabolite |
|  | Glycerolipid metabolism | Involved in inflammation in CF |
|  | Glycerophospholipid metabolism | Involved in inflammation in CF |
|  | Ether lipid metabolism | One or more Carbons on glycerol is bound to an alkyl chain via an ether linkage rather than the usual ester linkage |
|  | Fc gamma R-mediated phagocytosis | Phagocytosis/Engulfment of cells mediated by the Fc domain on certain cellular proteins |
|  | Fat digestion and absorption | Degradation and absorption of fats |
|  | Fatty acid biosynthesis | Fatty acid biosynthesis |
|  | AMPK signaling pathway | Cellular homeostasis – phage utilizes to enhance its survival |
|  | Insulin signaling pathway | Insulin |
|  | Lysine degradation | Lysine degrades DNA |
|  | Phenylalanine metabolism | Amino acid metabolism |
|  | Tryptophan metabolism | Amino acid metabolism |
|  | beta - Alanine metabolism | Amino acid metabolism |
|  | Aminobenzoate degradation | Aminobenzonate has been trialed as a drug previously |
|  | Proponate metabolism | Precursor to propanoic acid |
|  | Butanoate metabolism | Precursor to butanoic acid |
|  | Limonene and pinene degradation | Bronchitis drug – phage can breakdown |
|  | Caprolactam degradation | Natural product that is the basis of some drugs – phage aid breakdown? |
|  | alpha-Linolenic acid metabolism | Essential Fatty Acid |
|  | Biosynthesis of unsaturated fatty acids | Unsaturated Fatty Acid synthesis |
|  | PPAR signaling pathway | Link to obesity/insulin etc. |
|  | cAMP signaling pathway | Energy generation |
|  | Peroxisome | Link to obesity/insulin etc. |
|  | Steroid biosynthesis | Steroid generation |
| **< 10 year clinical diagnosis nCFBR** | Ether lipid metabolism | One or more Carbons on glycerol is bound to an alkyl chain via an ether linkage rather than the usual ester linkage |
|  | Fatty acid biosynthesis | Biosynthesis of fatty acids |
|  | Glycerophospholipid metabolism | Involved in inflammation in chronic lung diseases |
|  | Fatty acid biosynthesis, initiation | Initiation of fatty acid biosynthesis |
|  | Fatty acid biosynthesis, elongation | Elongation stage of fatty acid biosynthesis |
|  | Fatty acid degradation | Degradation of fatty acids |
|  |  |  |
| **Adult CF** | Sphingolipid metabolism | Involved in inflammation in CF |
|  | Taurine and hypotaurine metabolism | Taurine is found in a large proportion of animal tissues, hypotaurine is an intermediate in this process |
|  | Cyanoamino acid metabolism | Amino acid metabolism – addition of a nitrile group to the front of the amino acid |
|  | Glutathione metabolism | Antioxidant that protects cells from the damage caused by ROS (Reactive Oxygen Species) |
|  | Arachidonic acid metabolism | Unsaturated fatty acid |
|  | Fatty acid degradation | Degradation of fatty acids |
|  | alpha-Linolenic acid metabolism | Alter the fatty acid in CF – have an effect on potential CF diet management |
|  | Fatty acid metabolism | Self-explanatory |
|  | Glycerolipid metabolism | Lipid metabolism |
|  | Glycerophospholipid metabolism | Involved in inflammation in CF |
|  | Fatty acid biosynthesis | Biosynthesis of fatty acids |
|  | AMPK signaling pathway | Cellular homeostasis – phage utilizes to enhance its survival |
|  | Insulin signaling pathway | Insulin |
|  | Steroid hormone biosynthesis | Synthesis of steroid hormone’s |
|  | Metabolism of xenobiotics by cytochrome P450 | Cytochrome P450’s are key in xenobiotic degradation |
|  | Chemical carcinogenesis | Chemicals that are capable of causing cancer |
|  | Valine, leucine and isoleucine degradation | These are previously seen to be elevated in the CF lung |
|  | Geraniol degradation | Geraniol (bacterial nutrient source) – phage utilize for energy |
|  | Lysine degradation | Lysine degrades DNA |
|  | Phenylalanine metabolism | Amino acid metabolism |
|  | Benzoate degradation | Utilize alternative Carbon sources – longevity |
|  | Tryptophan metabolism | Amino acid metabolism |
|  | beta-Alanine metabolism | Amino acid metabolism |
|  | Aminobenzoate degradation | Aminobenzonate has been trialed as a drug previously |
|  | Propanoate metabolism | Precursor to propanoic acid |
|  | Butanoate metabolism | Precursor to butanoic acid |
|  | Limonene and pinene degradation | Drug degradation – bronchitis drug |
|  | Caprolactam degradation | Natural product that is the basis of some drugs |
|  | Synthesis and degradation of ketone bodies | Water soluble molecules produced in times of starvation, produced from Acetyl CoA |
|  | Carbon fixation pathways in Prokaryotes | Fixation of carbon in Prokaryotes |
|  | Terpenoid backbone biosynthesis | Secondary metabolite biosynthesis |
|  | Ethylbenzene degradation | Utilize alternative Carbon sources – longevity |
|  | Ether lipid metabolism | One or more Carbons on glycerol is bound to an alkyl chain via an ether linkage rather than the usual ester linkage |
| **> 10 year clinical diagnosis nCFBR** | Fatty acid degradation | Degradation of fatty acids |
|  | alpha-Linolenic acid metabolism | Alter the fatty acids in CF – have an effect on potential CF diet management |
|  | Glycerophospholipid metabolism | Involved in inflammation in chronic lung diseases |
|  | Ether lipid metabolism | One or more Carbons on glycerol is bound to an alkyl chain via an ether linkage rather than the usual ester linkage |
|  | Ras signaling pathway | Control of cellular signaling, normally involved in pathways that are involved in cell growth, differentiation and survival |
|  | cAMP signaling pathway | Energy generation |
|  | Endocytosis | Process where the cell engulfs products such as proteins, uses energy |
|  | Fc gamma R-mediated phagocytosis | Phagocytosis/Engulfment of cells mediated by the Fc domain on certain cellular proteins |
|  | Glutamatergic synapse | Post synaptic synapse – NMDA type |
|  | GnRH signaling pathway | Gonadotropin-releasing hormone generated (benzene ring product) |
|  | Sphingolipid metabolism | Involved in inflammation in chronic respiratory diseases |
|  | Phosphonate and phosphinate metabolism | Degradable organic Carbon products which can be used for pest control – benzene ring containing |
|  | Fatty acid biosynthesis | Biosynthesis of fatty acid’s |
|  | Fatty acid metabolism | Metabolism of fatty acid’s |
|  | AMPK signaling pathway | Cellular homeostasis – phage utilizes in order to enhance its survival |
|  | Insulin signaling pathway | Insulin productio |
|  | Valine, leucine and isoleucine degradation | These are seen to be elevated in the CF lung |
|  | Geraniol degradation | Geraniol (bacterial nutrient source) |
|  | Lysine degradation | Lysine degrades DNA |
|  | Phenylalanine metabolism | Amino acid metabolism |
|  | Benzoate degradation | Utilize alternative Carbon sources – longevity Phage encoded |
|  | Tryptophan metabolism | Amino acid metabolism |
|  | beta-Alanine metabolism | Amino acid metabolism |
|  | Aminobenzoate degradation | Aminobenzoate has been trialed as a drug previously |
|  | Propanoate metabolism | Precursor to propanoic acid |
|  | Butanoate metabolism | Precursor to butanoic acid |
|  | Limonene and pinene degradation | Bronchitis drug – phage can breakdown |
|  | Caprolactam degradation | Natural product that is the basis of some drugs – phage aid breakdown |
|  | Lipopolysaccharide biosynthesis | Cell wall |
|  | Steroid hormone biosynthesis | Synthesis of steroid hormone’s |
|  | Metabolism of xenobiotics by cytochrome P45 | Using cytochrome P45 to generate xenobiotics |
|  | Chemical carcinogenesis | Chemicals that are capable of causing cancer |
| **Metabolism of Terpenoids and Polyketides (secondary metabolites naturally produced)** | **KEGG Identification** | **Putative function** |
| **Pediatric CF** | Bisphenol degradation | Common chemical exposed to in daily life |
|  | Polycyclic aromatic hydrocarbon degradation | Break down of compounds containing these rings |
|  | Aminobenzoate degradation | Drug degradation |
|  | Limonene and pinene degradation | Drug degradation – bronchitis drug |
|  | Stilbenoid, diarylheptanoid and gingerol biosynthesis | Plant related drug |
| **< 10 year clinical diagnosis nCFBR** | Zeatin biosynthesis | Benzene ring plant hormone |
|  | Diterpenoid biosynthesis | Benzene ring plant hormone |
|  | Biosynthesis of secondary metabolites | Generation of secondary metabolites |
| **Adult CF** | Brassinosteroid biosynthesis | Plant hormone with a potential role in agricultural processes |
|  | Terpenoid backbone biosynthesis | Secondary metabolite biosynthesis |
| **> 10 year clinical diagnosis nCFBR** | Diterpenoid biosynthesis | Potential use for antibacterial functions, plant based metabolite |
|  | Brassinosteroid biosynthesis | Plant hormone with a potential role in agricultural processes |
| **CARBOHYDRATE METABOLISM** | **KEGG Identification** | **Putative function** |
| **Pediatric CF** | Galactose metabolism | Energy - sugar |
|  | Other glycan degradation | Chemicals that are capable of causing cancer |
|  | Glycosaminoglycan degradation | Long unbranched polysaccharides made of repeating disaccharide units |
|  | Sphingolipid metabolism | Involved in inflammation in CF |
|  | Glycosphingolipid biosynthesis - ganglio series | Involved in inflammation in CF |
|  | Lysosome | Break down of biomolecules including proteins, amino acid, lipids and CHO – found in animal cells |
|  | Phosphotransferase system (PTS) | Sugar transport system |
|  | Pentose and glucuronate interconversions | Transferring sugar groups |
|  | Fructose and mannose metabolism | Amino acid metabolism |
|  | Glycerolipid metabolism | Plant originating metabolite |
|  | Bisphenol degradation | Common chemical exposed to in daily life |
|  | Linoleic acid metabolism | Unsaturated fatty acid |
|  | Chloroalkane and chloroalkene degradation | Degrading chloro derivative alkene’s and alkane’s |
|  | Butanoate metabolism | Precursor to butanoic acid |
|  | Steroid hormone biosynthesis | Biosynthesis of steroid hormone’s |
|  | Metabolism of xenobiotics by cytochrome P450 | Cytochrome P450’s are key in xenobiotic degradation |
|  | Chemical carcinogenesis | Chemicals that are capable of causing cancer |
|  | Methane metabolism | Metabolism of methane |
|  | Carbon metabolism | Metabolism of carbon |
|  | Carbon fixation in photosynthetic organisms | Transfer of inorganic Carbon to organic Carbon |
|  | Glyoxylate and dicarboxylate metabolism | Reactions involving glyoxylate and dicarboxylate |
|  | Glycine, serine and threonine metabolism | Amino acid metabolism |
|  | Alanine, aspartate and glutamate metabolism | Amino acid metabolism |
|  | Peroxisome | Break down of long chain fatty acids by beta oxidation |
|  | Citrate cycle (TCA cycle) | Energy generation via the oxidization of acetate |
|  | Glutathione metabolism | Antioxidant that protects cells from the damage caused by ROS (Reactive Oxygen Species) |
|  | 2-Oxocarboxylic acid metabolism | The most elementary set of metabolites -pyruvate (2-oxopropanoate), 2-oxobutanoate, oxaloacetate (2-oxosuccinate) and 2-oxoglutarate |
|  | Biosynthesis of amino acids | Amino acid biosynthesis |
|  | Glycolysis / Gluconeogenesis | Converts glucose to pyruvate/Generation of glucose from non-Carbon sources |
|  | Fatty acid degradation | Degradation of fatty acid’s |
|  | Valine, leucine and isoleucine degradation | These are previously seen to be elevated in the CF lung |
|  | Lysine degradation | Lysine degrades DNA |
|  | Arginine and proline metabolism | Amino acid metabolism |
|  | Histidine metabolism | Amino acid metabolism |
|  | Tryptophan metabolism | Amino acid metabolism |
|  | beta-Alanine metabolism | Amino acid metabolism |
|  | Pyruvate metabolism | Glycolysis end product |
|  | Limonene and pinene degradation | Bronchitis drug – phage can breakdown |
|  | Propanoate metabolism | Precursor to propanoic acid |
|  | GABAergic synapse | Neurotransmitter in CNS (Central Nervous System) |
|  | Pentose phosphate pathway | Parallel to glycolysis and generates NAPDH and pentose |
|  | Ascorbate and aldarate metabolism | Ascorbate – Vitamin C by-product  Aldarate – acid generated from oxidation of the terminal groups on aldose |
|  | Caprolactam degradation | Natural product that is the basis of some drugs |
|  | Cyanoamino acid metabolism | Amino acid metabolism – addition of a nitrile group to the front of the amino acid |
|  | Tryptophan metabolism | Amino acid metabolism |
|  | Sulphur metabolism | Metabolism of sulphur |
|  | Cysteine and methionine metabolism | Metabolism of cysteine and methionine |
|  | Taurine and hypotaurine metabolism | Taurine is found in a large proportion of animal tissues, hypotaurine is an intermediate in this process |
| **< 10 year clinical diagnosis nCFBR** | Fructose and mannose metabolism | Amino acid metabolism |
|  | Glycolysis | Converts glucose to pyruvate |
|  | Nucleotide sugar biosynthesis | Biosynthesis of nucleotide sugars |
|  | Streptomycin biosynthesis | Antimicrobial – protein synthesis inhibitor - Aminoglycoside |
|  | Polyketide sugar unit biosynthesis | Secondary metabolites produced by most living organisms – contain a benzene ring |
|  | Lipopolysaccharide biosynthesis | Cell wall |
|  | Gluconeogenesis | Generation of glucose from non-Carbon sources |
|  | Pentose phosphate pathway | Parallel to glycolysis and generates NAPDH and pentose |
|  | Glyoxylate and dicarboxylate metabolism | Reactions involving glyoxylate and dicarboxylate |
| **Adult CF** | Galactose metabolism | Energy – sugar |
|  | Other glycan degradation | Degradation of glycan’s |
|  | Glycosaminoglycan degradation | Long unbranched polysaccharides made of repeating disaccharide units |
|  | Sphingolipid metabolism | Involved in inflammation CF |
|  | Glycosphingolipid biosynthesis - ganglio series | Involved in inflammation CF |
|  | Lysosome | Break down of biomolecules including proteins, amino acid, lipids and CHO – found in animal cells |
|  | Starch and sucrose metabolism | Energy generation |
|  | Fructose and mannose metabolism | Amino acid metabolism |
|  | Glycolysis / Gluconeogenesis | Converts glucose to pyruvate/Generation of glucose from non-Carbon sources |
|  | Amino sugar and nucleotide sugar metabolism | Generation of components of nucleic acids |
|  | Streptomycin biosynthesis | Antimicrobial – protein synthesis inhibitor - Aminoglycoside |
|  | Butirosin and neomycin biosynthesis | Aminoglycosides – drugs that originate from bacterial species |
|  | Carbon metabolism | Metabolism of carbon |
|  | HIF-1 signaling pathway | Cellular signaling - participate in angiogenesis, iron metabolism, glucose metabolism, and cell proliferation/survival |
|  | Carbohydrate digestion and absorption | Digestion and absorption of carbohydrate’s |
|  | Pyruvate metabolism | End product of glycolysis |
|  | Methane metabolism | Methane metabolism |
|  | Carbon fixation in photosynthetic organisms | Fixing carbon in photosynthetic organisms |
|  | Carbon fixation pathways in Prokaryotes | Fixation of carbon in Prokaryotes |
|  | Glyoxylate and dicarboxylate metabolism | Reactions involving glyoxylate and dicarboxylate |
|  | Pentose phosphate pathway | Parallel to glycolysis and generates NAPDH and pentose |
|  | Biosynthesis of ansamycins | Secondary metabolite – benzene ring product  Antimicrobial activity |
|  | Biosynthesis of amino acids | Synthesis of amino acids |
|  | Glycine, serine and threonine metabolism | Amino acid metabolism |
|  | Glycerolipid metabolism | Lipid metabolism |
|  | Bisphenol degradation | Common chemical exposed to in daily life |
|  | Linoleic acid metabolism | Unsaturated fatty acid |
|  | Chloroalkane and chloroalkene degradation | Degrading chloro derivative alkene’s and alkane’s |
|  | Butanoate metabolism | Precursor to butanoic acid |
|  | Pentose and glucuronate interconversions | Transferring sugar groups |
|  | Ascorbate and aldarate metabolism | Ascorbate – Vitamin C by-product  Aldarate – acid generated from oxidation of terminal groups on aldose |
|  | Fatty acid degradation | Degradation of fatty acid’s |
|  | Valine, leucine and isoleucine degradation | These are seen to be elevated in the CF lung |
|  | Lysine degradation | Lysine degrades DNA |
|  | Arginine and proline metabolism | Amino acid metabolism |
|  | Histidine metabolism | Amino acid metabolism |
|  | Tryptophan metabolism | Amino acid metabolism |
|  | beta-Alanine metabolism | Amino acid metabolism |
|  | Glycerolipid metabolism | Lipid metabolism |
|  | Limonene and pinene degradation | Bronchitis drug – phage can breakdown |
|  | Citrate cycle (TCA cycle) | Energy generation via the oxidation of acetate |
|  | Benzoate degradation | Utilize alternative Carbon sources – longevity |
|  | Bisphenol degradation | Common chemical exposed to in daily life |
|  | Naphthalene degradation | Double benzene ring product – used in mothballs |
|  | Butanoate metabolism | Precursor to butanoic acid |
|  | 2-Oxocarboxylic acid metabolism | The most elementary set of metabolites – pyruvate, oxaloacetate and 2 – oxoglutarate |
|  | C5-Branched dibasic acid metabolism | Involved in acid/base reactions |
| **> 10 year clinical diagnosis nCFBR** | Mannosyl phosphate transferase | Important in human metabolism – energy |
|  | Galactose metabolism | Energy metabolism |
|  | Other glycan degradation | Glycan degradation |
|  | Glycosaminoglycan degradation | Long unbranched polysaccharides made of repeating disaccharide units |
|  | Sphingolipid metabolism | Involved in inflammation in chronic respiratory diseases |
|  | Glycosphingolipid biosynthesis - ganglio series | Involved in inflammation in chronic respiratory diseases |
|  | Lysosome | Break down of biomolecules including proteins, amino acid, lipids and carbohydrates – found in animal cells |
|  | Pentose phosphate pathway | Parallel to glycolysis and generates NEPDH and pentose |
|  | Glutathione metabolism | Antioxidant that protects cells from the damage caused by ROS (Reactive Oxygen Species) |
|  | Carbon metabolism | Carbon metabolism |
|  | Central Carbon metabolism in cancer | Carbon metabolism in cancer |
|  | Pentose and glucuronate interconversions | Transferring sugar groups |
|  | Ascorbate and aldarate metabolism | Ascorbate – Vitamin C by-product  Aldarate – acid generated from oxidation of terminal groups on aldose |
|  | Starch and sucrose metabolism | Sugar metabolism |
|  | Amino sugar and nucleotide sugar metabolism | Generation of components of nucleic acids |
|  | Glycolysis / Gluconeogenesis | Converts glucose to pyruvate/Generation of glucose from non-Carbon sources |
|  | Fructose and mannose metabolism | Amino acid metabolism |
|  | Galactose metabolism | Energy – sugar |
|  | Streptomycin biosynthesis | Antimicrobial – protein synthesis inhibitor - Aminoglycoside |
|  | Butirosin and neomycin biosynthesis | Aminoglycosides – drugs that originate from bacterial species |
|  | HIF-1 signaling pathway | Cellular signaling - participate in angiogenesis, iron metabolism, glucose metabolism, and cell proliferation/survival |
|  | Insulin signaling pathway | Insulin production |
|  | Type II diabetes mellitus | Related to insulin |
|  | Carbohydrate digestion and absorption | Digestion and absorption of carbohydrates |
|  | Alanine, aspartate and glutamate metabolism | Amino acid metabolism |
|  | Cysteine and methionine metabolism | Amino acid metabolism |
|  | Sulphur metabolism | Metabolism of sulphur |
|  | Carbon metabolism | Metabolism of carbon |
|  | Biosynthesis of amino acids | Biosynthesis of amino acids |
|  | Histidine metabolism | Amino acid metabolism |
|  | Fatty acid degradation | Degradation of fatty acids |
|  | Valine, leucine and isoleucine degradation | These are seen to be elevated in the CF lung |
|  | Lysine degradation | Lysine degrades DNA |
|  | Arginine and proline metabolism | Amino acid metabolism |
|  | Histidine metabolism | Amino acid metabolism |
|  | Tryptophan metabolism | Amino acid metabolism |
|  | beta-Alanine metabolism | Amino acid metabolism |
|  | Glycerolipid metabolism | Lipid metabolism |
|  | Pyruvate metabolism | End product of glycolysis |
|  | Chloroalkane and chloroalkene degradation | Degrading chloro derivative alkene’s and alkane’s |
|  | Limonene and pinene degradation | Bronchitis drug – phage can breakdown |
|  | aldehyde dehydrogenase | Oxidise aldehyde’s |
|  | Glyoxylate and dicarboxylate metabolism | Reactions involving glyoxylate and dicarboxylate |
|  | Citrate cycle (TCA cycle) | Energy generation via the oxidation of acetate |
|  | Carbon fixation in photosynthetic organisms | Fixation of carbon in photosynthetic organisms |
|  | Methane metabolism | Metabolism of methane |
|  | Carbon fixation pathways in prokaryotes | Fixation of carbon in Prokaryotes |
|  | Benzoate degradation | Utilize alternative Carbon sources – longevity |
|  | Bisphenol degradation | Common chemical exposed to in daily life |
|  | Naphthalene degradation | Double benzene ring product – used in mothballs |
|  | Butanoate metabolism | Precursor to butanoic acid |
| **NUCLEOTIDE METABOLISM** | **KEGG Identification** | **Putative function** |
| **Pediatric CF** | Pyrimidine metabolism | Nucleotide protein – Cytosine/Thymine/Uracil |
|  | beta-Alanine metabolism | Amino acid metabolism |
|  | Pantothenate and CoA biosynthesis | Pantothenate is needed in animals to combine with CoA  CoA is involved in fatty acid metabolism and the TCA cycle |
|  | Drug metabolism - other enzymes | Drug metabolism |
|  | Purine metabolism | Nucleotide protein – Adenine/Guanine |
|  | DNA replication | Replication of DNA |
|  | Base excision repair | Involved in Nucleotide Excision Repair |
|  | Nucleotide excision repair | Removal of large damaged amino acids before mutations form |
|  | Homologous recombination | Repairs DSB (Double Strand Breaks) |
|  | Riboflavin metabolism | Vitamin B2, important cofactor in FAD which are essential in pathways such as - fatty acid metabolism, citrate cycle and electron transport chain |
|  | Nicotinate and nicotinamide metabolism | Nicotinamide (B vitamin) is the amide of nicotinic acid which is an essential human nutrient |
|  | Tyrosine metabolism | Amino acid metabolism |
|  | Biotin metabolism | B vitamin, involved in Gluconeogenesis |
| **< 10 year clinical diagnosis nCFBR** | Pyrimidine degradation | Degrading nucleotide proteins – Cytosine/Thymine/Uracil |
|  | Purine metabolism | Nucleotide protein metabolism – Adenine/Guanine |
|  | Pyrimidine metabolism | Metabolizing nucleotide proteins – Cytosine/Thymine/Uracil |
|  | DNA replication | Replication of DNA |
|  | Base excision repair | Involved in Nucleotide Excision Repair |
|  | Nucleotide excision repair | Removal of large damaged amino acids before mutations form |
|  | Homologous recombination | Repairs DSB (Double Strand Breaks) |
|  | Tyrosine metabolism | Amino acid metabolism |
|  | Tryptophan biosynthesis | Amino acid metabolism |
|  | Tyrosine degradation | Amino acid degradation |
|  | Phenylalanine metabolism | Amino acid metabolism |
|  | Glycine, serine and threonine metabolism | Amino acid metabolism |
|  | Cysteine and methionine metabolism | Amino acid metabolism |
|  | Arginine and proline metabolism | Amino acid metabolism |
|  | Glutathione metabolism | Antioxidant that protects cells from the damage caused by ROS (Reactive Oxygen Species) |
|  | Alanine, aspartate and glutamate metabolism | Amino acid metabolism |
|  | Nitrogen metabolism | Part of the nitrogen cycle – energy for the cell |
| **Adult CF** | Tyrosine metabolism | Amino acid metabolism |
|  | Styrene degradation | Benzene ring structure |
|  | Alanine, aspartate and glutamate metabolism | Amino acid metabolism |
|  | Arginine and proline metabolism | Amino acid metabolism |
|  | Glyoxylate and dicarboxylate metabolism | Reactions involving glyoxylate and dicarboxylate |
|  | Nitrogen metabolism | Nitrogen metabolism |
|  | Biosynthesis of amino acids | Amino acid biosynthesis |
|  | Two-component system | Response to the environment, allows adaptation to changing environments |
|  | Glutamatergic synapse | Post synaptic synapse – NMDA type |
|  | GABAergic synapse | Neurotransmitter in CNS (Central Nervous System) |
|  | Pyrimidine metabolism | Amino acid metabolism |
|  | beta-Alanine metabolism | Amino acid metabolism |
|  | Pantothenate and CoA biosynthesis | Pantothenate is needed in animals to combine with CoA  CoA is involved in fatty acid metabolism and the TCA cycle |
|  | Drug metabolism - other enzymes | Drug metabolism |
|  | Biotin metabolism | B vitamin, involved in Gluconeogenesis |
|  | Carbapenem biosynthesis | Beta lactam antibiotic |
|  | 2-Oxocarboxylic acid metabolism | The most elementary set of metabolites – pyruvate, oxaloacetate and 2 – oxoglutarate |
|  | Glutathione metabolism | Antioxidant that protects cells from the damage caused by ROS (Reactive Oxygen Species) |
|  | Cysteine and methionine metabolism | Amino acid metabolism |
|  | Valine, leucine and isoleucine degradation | These are seen to be elevated in the CF lung – possible phage degradation to increase survival |
|  | Histidine metabolism | Amino acid metabolism |
|  | Phenylalanine, tyrosine and tryptophan biosynthesis | Amino acid metabolism |
|  | Pyrimidine metabolism | Metabolizing nucleotide proteins – Cytosine/Thymine/Uracil |
|  | Selenocompound metabolism | Common metabolite found in a range of organisms |
|  | Phenylalanine metabolism | Amino acid metabolism |
|  | Purine metabolism | Nucleotide protein – Adenine/Guanine |
| **> 10 year clinical diagnosis nCFBR** | Purine metabolism | Nucleotide protein – Adenine/Guanine |
|  | Pyrimidine metabolism | Nucleotide protein – Cytosine/Thymine/Uracil |
|  | DNA replication | Replication of DNA |
|  | Base excision repair | Involved in Nucleotide Excision Repair |
|  | Nucleotide excision repair | Removal of large damaged amino acids before mutations form |
|  | Homologous recombination | Repairs DSB (Double Strand Breaks) |
|  | beta-Alanine metabolism | Amino acid metabolism |
|  | Pantothenate and CoA biosynthesis | Pantothenate is needed in animals to combine with CoA  CoA is involved in fatty acid metabolism and the TCA cycle |
|  | Drug metabolism - other enzymes | Drug metabolism |
| **ENERGY METABOLISM** | **KEGG Identification** | **Putative function** |
| **Pediatric CF** | Oxidative phosphorylation | Release of energy from the mitochondria |
|  | Porphyrin and chlorophyll metabolism | Porphyrin (Heme) and Chlorophyll are energy supplies |
| **< 10 year clinical diagnosis nCFBR** | Oxidative phosphorylation | Release of energy from the mitochondria |
|  | Porphyrin and chlorophyll metabolism | Porphyrin (Heme) and Chlorophyll are energy supplies |
|  | Cytochrome bc1 complex respiratory unit | Component in the respiratory chain complex – contains two heme groups and works in electron donating |
|  | Pyruvate metabolism | Glycolysis end product |
|  | Carbon fixation in photosynthetic organisms | Transfer of inorganic Carbon to organic Carbon |
|  | Carbon metabolism | Metabolism of Carbon |
|  | Glyoxylate and dicarboxylate metabolism | Reactions involving glyoxylate and dicarboxylate |
|  | Methane metabolism | Methane metabolism |
|  | Carbon metabolism | Methane metabolism |
| **Adult CF** | Oxidative phosphorylation | Release of energy from the mitochondria |
|  |  |  |
| **> 10 year clinical diagnosis nCFBR** | Galactose metabolism | Energy – sugar |
|  | Other glycan degradation | Glycan degradation |
|  | Sphingolipid metabolism | Involved in inflammation in chronic lung diseases |
|  | Starch and sucrose metabolism | Sugar metabolism |
|  | F-type ATPase | Energy generation – ATP |
|  | Oxidative phosphorylation | Release of energy from the mitochondria |
|  | Photosynthesis | Energy supply generated by the mitochondria |
| **XENOBIOTIC DEGRADATION** | **KEGG Identification** | **Putative function** |
| **Pediatric CF** | Caprolactam degradation | Natural product that is the basis of some drugs |
|  | Dioxin degradation | Heterocyclic 6 membered ring where two Carbons are replaced by oxygen |
|  | Degradation of aromatic compounds | Degradation of amino acids |
|  | Benzene degradation | Benzene ring degradation |
|  | Chlorocyclohexane and chlorobenzene degradation | Chlorobenzene – common chemical, initially DTT (pesticide) |
|  | Fluorobenzoate degradation | Utilize alternative Carbon sources – longevity |
|  | Toluene degradation | Benzene ring derivative – drug related |
|  | Degradation of aromatic compounds | Degradation of aromatic compounds |
|  | Polycyclic aromatic hydrocarbon degradation | Degradation of polycyclic hydrocarbons |
| **< 10 year clinical diagnosis nCFBR** | Benzoate degradation | Utilize alternative Carbon sources – longevity |
|  | Dioxin degradation | Heterocyclic 6 membered ring where two Carbons are replaced by oxygen |
|  | Toluene degradation | Benzene ring derivative - drug related |
|  | Degradation of aromatic compounds | Degradation of aromatic compounds |
| **Adult CF** | Toluene degradation | Benzene ring derivative - drug related |
|  | Degradation of aromatic compounds | Degradation of aromatic compounds |
|  | Polycyclic aromatic hydrocarbon degradation | Degradation of polycyclic hydrocarbons |
|  | Benzoate degradation | Utilize alternative Carbon sources – longevity |
|  | Chlorocyclohexane and chlorobenzene degradation | Chlorobenzene – common chemical, initially DTT (pesticide) |
|  | Bisphenol degradation | Common chemical exposed to in daily life |
|  | Naphthalene degradation | Double benzene ring product – used in mothballs |
|  | Aminobenzoate degradation | Aminobenzoate has been trialled as a drug previously |
|  | Limonene and pinene degradation | Bronchitis drug – phage can breakdown |
|  | Caprolactam degradation | Natural product that is the basis of some drugs |
|  | Drug metabolism - other enzymes | Drug metabolism |
| **> 10 year clinical diagnosis nCFBR** | Chlorocyclohexane and chlorobenzene degradation | Benzene ring degradation |
|  | Chloroalkane and chloroalkene degradation | Chlorobenzene – common chemical, initially DTT (pesticide) |
|  | Atrazine degradation | Degrading chloro derivative alkene’s and alkane’s |
|  | Caprolactam degradation | Herbicide – benzene ring product |
|  | Toluene degradation | Benzene ring derivative - drug related |
|  | Degradation of aromatic compounds | Degradation of aromatic compounds |
|  |  |  |
| **MECHANISM OF COFACTORS AND VITAMINS** | **KEGG Identification** | **Putative function** |
| **Pediatric CF** | N/A | |
| **< 10 year clinical diagnosis nCFBR** | N/A | |
| **Adult CF** | Ubiquinone and other terpenoid-quinone biosynthesis | Involvement in QS (Quorum Sensing) |
|  | Thiamine metabolism | B vitamin – Benzene ring product |
|  | Sulphur relay system | Generation of sulphur – ubiquinated products aid its movement |
|  | Riboflavin metabolism | Vitamin B2, important cofactor in FAD which are essential in pathways such as - fatty acid metabolism, citrate cycle and electron transport chain |
|  | Porphyrin and chlorophyll metabolism | Porphyrin (Heme) and Chlorophyll are energy supplies |
| **> 10 year clinical diagnosis nCFBR** | Thiamine metabolism | B vitamin – Benzene ring product |
|  | Sulphur relay system | Generation of sulphur – ubiquinated products aid its movement |
| **METABOLISM OF OTHER AMINO ACIDS** | **KEGG Identification** | **Putative function** |
| **Pediatric CF** | Arginine and proline metabolism | Amino acid metabolism |
|  | Glutathione metabolism | Antioxidant that protects cells from the damage caused by ROS (Reactive Oxygen Species) |
|  | Lysine biosynthesis | DNA degradation enzyme |
|  | Lysine degradation | Lysine degrades DNA |
|  | Biosynthesis of amino acids | Biosynthesis of amino acids |
|  | Valine, leucine and isoleucine degradation | These are previously seen to be elevated in the CF lung |
|  | Glycine, serine and threonine metabolism | Amino acid metabolism |
|  | Cysteine and methionine metabolism | Amino acid metabolism |
|  | 2-Oxocarboxylic acid metabolism | The most elementary set of metabolites -pyruvate (2-oxopropanoate), 2-oxobutanoate, oxaloacetate (2-oxosuccinate) and 2-oxoglutarate |
|  | Pantothenate and CoA biosynthesis | Pantothenate is needed in animals to combine with CoA  CoA is involved in fatty acid metabolism and the TCA cycle |
|  | Valine, leucine and isoleucine biosynthesis | Amino acid metabolism – seen in elevated levels in CF lungs |
|  | Alanine, aspartate and glutamate metabolism | Amino acid metabolism |
|  | Two-component system | Response to the environment, allows adaptation to changing environments |
|  | Glutamatergic synapse | Post synaptic synapse – NMDA type |
|  | GABAergic synapse | Neurotransmitter in CNS (Central Nervous System) |
| **< 10 year clinical diagnosis nCFBR** | N/A | |
| **Adult CF** | Lysine biosynthesis | DNA degradation enzyme |
|  | Peptidoglycan biosynthesis | Sugar metabolism in the cytosol – various enzymes needed |
|  | Histidine metabolism | Amino acid metabolism |
|  | Phenylalanine metabolism | Amino acid metabolism |
|  | Arginine and proline metabolism | Amino acid metabolism |
|  | Taurine and hypotaurine metabolism | Taurine is found in a large proportion of animal tissues, hypotaurine is an intermediate in this process |
|  | Cyanoamino acid metabolism | Amino acid metabolism – addition of a nitrile group to the front of the amino acid |
|  | Glutathione metabolism | Antioxidant that protects cells from the damage caused by ROS (Reactive Oxygen Species) |
|  | Aminoacyl-tRNA biosynthesis | Combines with amino acid whilst they are being synthesized |
|  | Alanine, aspartate and glutamate metabolism | Amino acid metabolism |
|  | Biosynthesis of amino acids | Biosynthesis of amino acids |
|  | Phenylalanine, tyrosine and tryptophan biosynthesis | Amino acid metabolism |
|  | Cysteine and methionine metabolism | Amino acid metabolism |
| **> 10 year clinical diagnosis nCFBR** | Lysine biosynthesis | DNA degradation enzyme |
|  | 2-Oxocarboxylic acid metabolism | The most elementary set of metabolites – pyruvate, oxaloacetate and 2 – oxoglutarate |
|  | Biosynthesis of amino acids | Biosynthesis of amino acids |
|  | Valine, leucine and isoleucine biosynthesis | These are seen to be elevated in the CF lung – possible phage degradation to increase survival |
|  | Pyruvate metabolism | End product of glycolysis |
|  | Arginine and proline metabolism | Amino acid metabolism |
|  | Tryptophan metabolism | Amino acid metabolism |
|  | Glutathione metabolism | Antioxidant that protects cells from the damage caused by ROS (Reactive Oxygen Species) |
| **BIOSYNTHESIS OF OTHER SECONDARY METABOLITES** | **KEGG Identification** | **Putative function** |
| **Pediatric CF** | N/A | |
| **< 10 year clinical diagnosis nCFBR** | Ubiquinone and other terpenoid-quinone biosynthesis | Involvement in QS (Quorum Sensing) |
|  | Porphyrin and chlorophyll metabolism | Porphyrin (Heme) and Chlorophyll are energy supplies |
|  | Drug metabolism - other enzymes | Drug metabolism |
|  | Lysine degradation | Lysine degrades DNA – possible survival tactic |
|  | Biotin metabolism | B vitamin, involved in Gluconeogenesis |
| **Adult CF** | N/A | |
| **> 10 year clinical diagnosis nCFBR** | Heme biosynthesis | Energy supply |
|  | Porphyrin and chlorophyll metabolism | Porphyrin (Heme) and Chlorophyll are energy supplies |
|  | Ubiquinone biosynthesis | Coenzyme Q10, benzene ring product |

**S3** – The raw data from MG RAST generated for all the samples which passed its internal quality control, 11/17 < 10 years since clinical diagnosis nCFBR, 25/28 > 10 years since clinical diagnosis nCFBR, 10/10 Pediatric CF and 36/37 Adult CF.

| **Clinical group** | **Sample ID** | **Number of Reads uploaded to MG-RAST** |
| --- | --- | --- |
| < 10 year nCFBR | Phage50 | 135,229 |
|  | Phage52 | 479,192 |
|  | Phage68 | 698,674 |
|  | Phage72 | 496,093 |
|  | Phage73 | 618,612 |
|  | Phage74 | 439,489 |
|  | Phage76 | 23,898 |
|  | Phage77 | 278,743 |
|  | Phage80 | 862,415 |
|  | Phage86 | 404,422 |
|  | Phage91 | 383,572 |
| > 10 year nCFBR | Phage46 | 25,262 |
|  | Phage47 | 397,339 |
|  | Phage48 | 546,201 |
|  | Phage51 | 248,902 |
|  | Phage53 | 313,599 |
|  | Phage54 | 372,256 |
|  | Phage55 | 904,450 |
|  | Phage56 | 258,012 |
|  | Phage59 | 368,431 |
|  | Phage60 | 39,831 |
|  | Phage61 | 382,264 |
|  | Phage62 | 274,339 |
|  | Phage63 | 362,896 |
|  | Phage64 | 326,612 |
|  | Phage65 | 1,018,124 |
|  | Phage69 | 415,920 |
|  | Phage75 | 97,351 |
|  | Phage78 | 708,098 |
|  | Phage79 | 442,845 |
|  | Phage85 | 236,330 |
|  | Phage87 | 17,953 |
|  | Phage88 | 540,788 |
|  | Phage89 | 1,115,698 |
|  | Phage92 | 594,975 |
|  | Phage93 | 62,894 |
| Pediatric CF | Phage13 | 606,526 |
|  | Phage14 | 232,852 |
|  | Phage22 | 26,409 |
|  | Phage23 | 17,862 |
|  | Phage32 | 308,499 |
|  | Phage40 | 710,926 |
|  | Phage43 | 1,020,308 |
|  | Phage44 | 337,907 |
|  | Phage45 | 24,446 |
|  | Phage83 | 15,484 |
| Adult CF | Phage1 | 336,053 |
|  | Phage2 | 10,860 |
|  | Phage3 | 1,112,907 |
|  | Phage4 | 46,152 |
|  | Phage5 | 335,623 |
|  | Phage6 | 453,949 |
|  | Phage7 | 307,972 |
|  | Phage8 | 649,773 |
|  | Phage9 | 809,956 |
|  | Phage10 | 25,305 |
|  | Phage12 | 57,446 |
|  | Phage15 | 371,506 |
|  | Phage16 | 806,898 |
|  | Phage17 | 343,106 |
|  | Phage18 | 983,127 |
|  | Phage19 | 1,035,849 |
|  | Phage20 | 238,769 |
|  | Phage21 | 1,092,624 |
|  | Phage24 | 19,468 |
|  | Phage25 | 129,064 |
|  | Phage26 | 93,154 |
|  | Phage27 | 90,725 |
|  | Phage28 | 978,403 |
|  | Phage29 | 256,542 |
|  | Phage30 | 56, 252 |
|  | Phage31 | 397,022 |
|  | Phage33 | 434,882 |
|  | Phage34 | 186,544 |
|  | Phage35 | 493,891 |
|  | Phage36 | 355,818 |
|  | Phage37 | 252,476 |
|  | Phage38 | 16,271 |
|  | Phage39 | 653,032 |
|  | Phage41 | 442,560 |
|  | Phage42 | 685,801 |
|  | Phage84 | 44,228 |

**S4** – A list of EC numbers derived in KEGG relating to our disease classification criteria (as described in the materials and methods) and the proposed metabolic pathways.

| **Pa Phages isolated from Patients < 10 year clinical diagnosis**  **nCFBR** | **KEGG EC number** | **Identified Pathway** |
| --- | --- | --- |
|  | 2.7.7.7 | Nucleotide metabolism |
|  | 3.6.4.12 | Nucleotide metabolism |
|  | 2.7.13.3 | Histidine kinase |
|  | 2.7.1.69 | Carbohydrate metabolism |
|  | 2.3.1.- | Biosynthesis of other secondary metabolites |
|  | 4.3.1.3 | Carbohydrate metabolism |
|  | 2.5.1.75 | Biosynthesis of other secondary metabolites |
|  | 4.1.3.27 | Biosynthesis of other secondary metabolites |
|  | 1.1.99.3 | Carbohydrate metabolism |
|  | 3.1.3.82 | Glycan biosynthesis and metabolism |
|  | 3.1.3.83 | Lipid metabolism |
|  | 3.5.1.96 | Nucleotide metabolism |
|  | 3.5.2.6 | Biosynthesis of other secondary metabolites |
|  | 1.8.1.2 | Carbohydrate metabolism |
|  | 2.1.1.13 | Biosynthesis of other secondary metabolites |
|  | 2.7.11.1 | Kinase |
|  | 3.1.11.5 | Hydrolases |
|  | 3.4.-.- | Hydrolases |
|  | 1.10.3.- | Biosynthesis of other secondary metabolites |
|  | 1.2.1.2 | Carbohydrate metabolism |
|  | 6.3.5.4 | Biosynthesis of other secondary metabolites |
|  | 5.1.3.15 | Biosynthesis of other secondary metabolites |
|  | 1.3.1.2 | Nucleotide metabolism |
|  | 1.4.1.2 | Nucleotide metabolism |
|  | 1.7.1.4 | Nucleotide metabolism |
|  | 1.4.3.- | Energy metabolism |
|  | 1.1.3.15 | Biosynthesis of other secondary metabolites |
|  | 4.1.1.17 | Biosynthesis of other secondary metabolites |
|  | 5.4.99.16 | Carbohydrate metabolism |
|  | 3.2.1.1 | Carbohydrate metabolism |
|  | 1.9.3.1 | Energy metabolism |
|  | 4.1.1.- | Biosynthesis of other secondary metabolites |
|  | 2.5.1.17 | Energy metabolism |
|  | 6.3.5.10 | Energy metabolism |
|  | 2.7.7.24 | Biosynthesis of other secondary metabolites |
|  | 2.7.2.2 | Nucleotide metabolism |
|  | 1.2.1.18 | Carbohydrate metabolism |
|  | 1.2.1.27 | Lipid metabolism |
|  |  |  |
| **Pa Phages isolated from Patients > 10 year clinical diagnosis**  **nCFBR** | **KEGG EC number** | **Identified Pathway** |
|  | 3.5.1.11 | Biosynthesis of other secondary metabolites |
|  | 3.6.4.12 | Nucleotide metabolism |
|  | 1.2.1.3 | Carbohydrate metabolism |
|  | 1.3.99.- | Lipid metabolism |
|  | 2.7.7.7 | Carbohydrate metabolism |
|  | 1.13.12.16 | Nucleotide metabolism |
|  | 1.3.8.7 | Lipid metabolism |
|  | 2.1.1.37 | Carbohydrate metabolism |
|  | 4.2.1.17 | Lipid metabolism |
|  | 2.5.1.18 | Lipid metabolism |
|  | 3.2.1.23 | Carbohydrate metabolism |
|  | 2.5.1.47 | Carbohydrate metabolism |
|  | 1.1.1.- | Lipid metabolism |
|  | 2.7.1.31 | Carbohydrate metabolism |
|  | 3.1.1.1 | Nucleotide metabolism |
|  | 1.6.5.3 | Energy metabolism |
|  | 1.1.1.100 | Lipid metabolism |
|  | 1.17.4.1 | Nucleotide metabolism |
|  | 1.9.3.1 | Energy metabolism |
|  | 2.7.13.3 | Histidine kinase |
|  | 3.5.1.- | Nucleotide metabolism |
|  | 1.4.3.- | Energy metabolism |
|  | 2.3.1.- | Biosynthesis of other secondary metabolites |
|  | 6.6.1.2 | Energy metabolism |
|  | 1.5.3.1 | Metabolism of other amino acids |
|  | 6.4.1.5 | Biosynthesis of other secondary metabolites |
|  | 3.6.3.- | ATPase |
|  | 3.1.4.3 | Lipid metabolism |
|  | 1.7.99.7 | Oxidoreductases |
|  | 1.5.99.6 | Metabolism of other amino acids |
|  | 1.17.1.1 | Biosynthesis of other secondary metabolites |
|  | 2.3.2.2 | Metabolism of other amino acids |
|  | 3.1.2.1 | Metabolism of other amino acids |
|  | 3.6.3.14 | Energy metabolism |
|  | 1.7.99.4 | Nucleotide metabolism |
|  | 3.5.3.11 | Nucleotide metabolism |
|  | 3.5.2.6 | Biosynthesis of other secondary metabolites |
|  | 3.8.1.3 | Xenobiotic degradation |
|  | 3.5.99.3 | Xenobiotic degradation |
|  | 3.1.4.46 | Lipid metabolism |
|  | 6.3.4.15 | Nucleotide metabolism |
|  | 1.2.1.2 | Carbohydrate metabolism |
|  | 3.6.3.41 | ATPase |
|  | 1.11.1.6 | Biosynthesis of other secondary metabolites |
|  | 2.3.1.179 | Nucleotide metabolism |
|  | 2.3.1.39 | Lipid metabolism |
|  | 2.4.1.18 | Biosynthesis of other secondary metabolites |
|  | 6.3.3.1 | Biosynthesis of other secondary metabolites |
|  | 6.4.1.2 | Lipid metabolism |
|  | 2.3.1.51 | Carbohydrate metabolism |
|  | 2.-.-.- | Unknown |
|  | 4.1.3.30 | Lipid metabolism |
|  | 2.4.1.129 | Carbohydrate metabolism |
|  | 3.4.-.- | Unknown |
|  | 1.1.1.49 | Carbohydrate metabolism |
|  | 2.7.2.2 | Nucleotide metabolism |
|  | 3.1.3.11 | Carbohydrate metabolism |
|  | 2.7.7.13 | Carbohydrate metabolism |
|  | 1.7.2.4 | Nucleotide metabolism |
|  | 6.4.1.1 | Carbohydrate metabolism |
|  | 4.1.1.75 | Carbohydrate metabolism |
|  | 1.13.11.27 | Lipid metabolism |
|  | 4.1.1.37 | Energy metabolism |
|  | 2.7.11.1 | Kinase |
|  | 5.1.3.20 | Lipid metabolism |
|  | 6.2.1.1 | Carbohydrate metabolism |
|  | 1.2.1.60 | Nucleotide metabolism |
|  | 1.10.3.- | Biosynthesis of other secondary metabolites |
|  | 1.13.11.15 | Nucleotide metabolism |
|  | 4.2.1.- | Nucleotide metabolism |
|  | 5.3.3.10 | Nucleotide metabolism |
|  | 3.4.11.1 | Nucleotide metabolism |
|  | 4.2.1.20 | Carbohydrate metabolism |
|  | 1.6.1.2 | Biosynthesis of other secondary metabolites |
|  | 2.6.1.62 | Nucleotide metabolism |
|  | 5.1.1.7 | Metabolism of other amino acids |
|  | 1.1.1.22 | Carbohydrate metabolism |
|  | 2.3.1.109 | Carbohydrate metabolism |
|  | 1.1.1.169 | Nucleotide metabolism |
|  | 4.1.1.48 | Nucleotide metabolism |
|  | 2.3.1.41 | Lipid metabolism |
|  | 1.1.1.94 | Lipid metabolism |
|  | 2.1.2.13 | Biosynthesis of other secondary metabolites |
|  | 1.1.1.305 | Biosynthesis of other secondary metabolites |
|  | 3.5.4.3 | Nucleotide metabolism |
|  | 2.1.2.9 | Metabolism of other amino acids |
|  | 2.6.1.87 | Biosynthesis of other secondary metabolites |
|  | 3.5.1.41 | Biosynthesis of other secondary metabolites |
|  | 3.1.11.5 | Hydrolase |
|  | 6.3.1.2 | Carbohydrate metabolism |
|  | 4.3.1.17 | Carbohydrate metabolism |
|  | 5.4.4.2 | Biosynthesis of other secondary metabolites |
|  | 1.1.1.35 | Lipid metabolism |
|  | 4.2.1.17 | Lipid metabolism |
|  | 5.1.2.3 | Lipid metabolism |
|  | 3.5.1.6 | Nucleotide metabolism |
|  | 3.5.1.32 | Nucleotide metabolism |
|  | 1.7.1.4 | Nucleotide metabolism |
|  | 4.2.1.47 | Carbohydrate metabolism |
|  | 1.4.4.2 | Carbohydrate metabolism |
|  | 3.5.2.3 | Nucleotide metabolism |
|  | 2.1.1.222 | Biosynthesis of other secondary metabolites |
|  | 2.1.1.64 | Biosynthesis of other secondary metabolites |
|  | 2.3.1.180 | Lipid metabolism |
|  | 5.4.99.16 | Carbohydrate metabolism |
|  | 2.2.1.1 | Carbohydrate metabolism |
|  | 2.2.1.6 | Metabolism of other amino acids |
|  | 3.5.3.8 | Carbohydrate metabolism |
|  | 3.5.1.10 | Carbohydrate metabolism |
|  | 3.4.11.5 | Carbohydrate metabolism |
|  | 1.1.2.3 | Carbohydrate metabolism |
|  | 3.1.3.16 | Cell signalling |
|  | 4.3.1.3 | Carbohydrate metabolism |
|  | 2.1.1.80 | Transferases |
|  | 2.3.3.13 | Lipid metabolism |
|  | 2.7.7.23 | Biosynthesis of other secondary metabolites |
|  | 2.3.1.157 | Carbohydrate metabolism |
|  | 6.1.1.5 | Metabolism of other amino acids |
|  | 3.5.3.6 | Metabolism of other amino acids |
|  | 2.3.1.31 | Carbohydrate metabolism |
|  | 1.14.12.1 | Xenobiotic degradation |
|  | 3.5.1.53 | Carbohydrate metabolism |
|  | 6.3.2.2 | Lipid metabolism |
|  | 3.5.1.44 | Hydrolases |
|  | 3.1.5.1 | Nucleotide metabolism |
|  | 5.3.1.8 | Carbohydrate metabolism |
|  | 1.1.99.1 | Carbohydrate metabolism |
|  | 4.2.1.9 | Lipid metabolism |
|  | 1.1.1.38 | Carbohydrate metabolism |
|  | 3.5.2.7 | Carbohydrate metabolism |
|  | 1.2.4.1 | Carbohydrate metabolism |
|  | 3.1.3.3 | Carbohydrate metabolism |
|  | 2.7.1.39 | Carbohydrate metabolism |
|  | 2.5.1.49 | Carbohydrate metabolism |
|  | 4.2.1.33 | Lipid metabolism |
|  | 4.2.1.35 | Lipid metabolism |
|  | 6.1.1.1 | Metabolism of other amino acids |
|  | 1.2.1.18 | Carbohydrate metabolism |
|  | 1.2.1.27 | Lipid metabolism |
|  | 2.7.8.- | Lipid metabolism |
|  | 3.6.1.54 | Glycan biosynthesis and metabolism |
|  |  |  |
| **Pa Phages isolated from Pediatric CF patients, <15 yr** | **KEGG EC number** | **Identified Pathway** |
|  | 3.6.4.12 | Nucleotide metabolism |
|  | 3.5.1.11 | Biosynthesis of other secondary metabolites |
|  | 2.7.7.7 | Nucleotide metabolism |
|  | 3.2.1.23 | Carbohydrate metabolism |
|  | 1.2.1.3 | Carbohydrate metabolism |
|  | 1.3.99.- | Lipid metabolism |
|  | 1.3.8.7 | Lipid metabolism |
|  | 1.13.12.16 | Nucleotide metabolism |
|  | 4.2.1.17 | Lipid metabolism |
|  | 2.5.1.47 | Carbohydrate metabolism |
|  | 2.5.1.18 | Lipid metabolism |
|  | 2.7.1.31 | Carbohydrate metabolism |
|  | 1.1.1.- | Lipid metabolism |
|  | 3.1.1.1 | Nucleotide metabolism |
|  | 1.6.5.3 | Energy metabolism |
|  | 4.2.1.3 | Carbohydrate metabolism |
|  | 4.2.1.- | Nucleotide metabolism |
|  | 2.6.1.18 | Lipid metabolism |
|  | 2.1.2.1 | Biosynthesis of other secondary metabolites |
|  | 2.7.1.35 | Mechanism of co factors and vitamins |
|  | 3.1.7.2 | Nucleotide metabolism |
|  | 6.1.1.9 | Metabolism of other amino acids |
|  | 1.9.3.1 | Energy metabolism |
|  | 1.1.1.100 | Lipid metabolism |
|  | 2.3.1.117 | Metabolism of other amino acids |
|  | 4.3.1.12 | Carbohydrate metabolism |
|  | 6.6.1.2 | Energy metabolism |
|  | 3.4.21.53 | Hydrolase |
|  | 3.3.2.1 | Biosynthesis of other secondary metabolites |
|  | 2.7.6.5 | Nucleotide metabolism |
|  | 2.3.1.57 | Carbohydrate metabolism |
|  | 2.4.2.11 | Transferase |
|  | 2.7.4.22 | Nucleotide metabolism |
|  | 3.4.11.2 | Biosynthesis of other secondary metabolites |
|  | 3.5.2.6 | Biosynthesis of other secondary metabolites |
|  | 2.1.1.163 | Biosynthesis of other secondary metabolites |
|  | 2.1.1.201 | Biosynthesis of other secondary metabolites |
|  | 1.14.14.9 | Nucleotide metabolism |
|  | 4.1.1.50 | Carbohydrate metabolism |
|  | 3.1.5.1 | Nucleotide metabolism |
|  | 2.1.1.107 | Biosynthesis of other secondary metabolites |
|  | 1.3.1.76 | Biosynthesis of other secondary metabolites |
|  | 4.99.1.4 | Biosynthesis of other secondary metabolites |
|  | 5.4.2.1 | Isomerase |
|  | 1.14.-.- | Lipid metabolism |
|  | 1.1.1.42 | Carbohydrate metabolism |
|  | 4.2.2.3 | Carbohydrate metabolism |
|  | 2.3.1.16 | Lipid metabolism |
|  | 2.1.1.13 | Biosynthesis of other secondary metabolites |
|  | 5.5.1.1 | Xenobiotic degradation |
|  | 1.13.11.3 | Lipid metabolism |
|  | 6.3.1.2 | Carbohydrate metabolism |
|  | 1.2.1.2 | Carbohydrate metabolism |
|  | 1.3.98.1 | Nucleotide metabolism |
|  | 3.5.3.13 | Carbohydrate metabolism |
|  | 3.5.1.6 | Nucleotide metabolism |
|  | 4.1.3.38 | Mechanisms of cofactors and vitamins |
|  | 4.2.1.47 | Carbohydrate metabolism |
|  | 2.1.1.222 | Biosynthesis of other secondary metabolites |
|  | 2.1.1.64 | Biosynthesis of other secondary metabolites |
|  | 2.3.1.- | Biosynthesis of other secondary metabolites |
|  | 2.7.7.4 | Nucleotide metabolism |
|  | 2.7.1.25 | Nucleotide metabolism |
|  | 1.2.1.46 | Carbohydrate metabolism |
|  | 2.7.13.3 | Histidine kinase |
|  | 5.3.1.22 | Carbohydrate metabolis |
|  | 1.1.99.3 | Carbohydrate metabolism |
|  | 1.2.1.11 | Carbohydrate metabolism |
|  | 1.8.1.2 | Carbohydrate metabolism |
|  | 3.4.-.- | Hydrolase |
|  | 4.2.1.9 | Lipid metabolism |
|  | 1.2.4.1 | Carbohydrate metabolism |
|  | 1.4.3.- | Energy metabolism |
|  | 3.1.3.- | Nucleotide metabolism |
|  | 3.1.1.3 | Lipid metabolism |
|  | 6.2.1.3 | Lipid metabolism |
|  | 2.4.1.129 | Carbohydrate metabolism |
|  | 3.5.4.4 | Nucleotide metabolism |
|  |  |  |
| **Pa Phages Isolated from Adult CF Patients >15yr** | **EC number** | **Function** |
|  | 3.5.1.11 | Biosynthesis of other secondary metabolites |
|  | 3.6.4.12 | Nucleotide metabolism |
|  | 1.2.1.3 | Carbohydrate metabolism |
|  | 1.3.99.- | Lipid metabolism |
|  | 1.13.12.16 | Nucleotide metabolism |
|  | 1.3.8.7 | Lipid metabolism |
|  | 2.7.7.7 | Nucleotide metabolism |
|  | 4.2.1.17 | Lipid metabolism |
|  | 3.2.1.23 | Carbohydrate metabolism |
|  | 2.5.1.18 | Lipid metabolism |
|  | 2.5.1.47 | Carbohydrate metabolism |
|  | 1.1.1.- | Lipid metabolism |
|  | 2.7.1.31 | Carbohydrate metabolism |
|  | 3.1.1.1 | Nucleotide metabolism |
|  | 1.6.5.3 | Energy metabolism |
|  | 3.6.3.- | ATPase |
|  | 2.7.11.1 | Kinase |
|  | 1.1.1.100 | Lipid metabolism |
|  | 3.1.5.1 | Nucleotide metabolism |
|  | 5.4.4.2 | Biosynthesis of other secondary metabolites |
|  | 1.2.1.18 | Carbohydrate metabolism |
|  | 1.2.1.27 | Lipid metabolism |
|  | 2.2.1.1 | Carbohydrate metabolism |
|  | 1.5.3.1 | Metabolism of other amino acids |
|  | 2.7.13.3 | Histidine kinase |
|  | 4.2.1.- | Nucleotide metabolism |
|  | 1.4.1.13 | Nucleotide metabolism |
|  | 1.4.1.14 | Nucleotide metabolism |
|  | 1.4.3.- | Energy metabolism |
|  | 1.1.1.38 | Carbohydrate metabolism |
|  | 3.6.3.14 | Energy metabolism |
|  | 2.7.1.69 | Carbohydrate metabolism |
|  | 1.2.1.2 | Carbohydrate metabolism |
|  | 3.6.3.31 | ATPase |
|  | 2.4.2.9 | Nucleotide metabolism |
|  | 1.9.3.1 | Energy metabolism |
|  | 4.2.1.18 | Lipid metabolism |
|  | 6.2.1.1 | Carbohydrate metabolism |
|  | 6.3.1.2 | Carbohydrate metabolism |
|  | 3.5.1.32 | Nucleotide metabolism |
|  | 5.4.99.16 | Carbohydrate metabolism |
|  | 3.2.1.1 | Carbohydrate metabolism |
|  | 1.7.99.7 | Oxidoreductases |
|  | 6.5.1.1 | Nucleotide metabolism |
|  | 1.14.14.9 | Nucleotide metabolism |
|  | 4.3.2.2 | Nucleotide metabolism |
|  | 6.4.1.4 | Lipid metabolism |
|  | 1.14.12.1 | Xenobiotic degradation |
|  | 2.3.2.2 | Metabolism of other amino acids |
|  | 6.1.1.18 | Metabolism of other amino acids |
|  | 3.1.2.1 | Metabolism of other amino acids |
|  | 3.4.21.53 | Hydrolase |
|  | 4.3.1.18 | Carbohydrate metabolism |
|  | 3.5.1.19 | Mechanisms of other co factors and vitamins |
|  | 3.5.1.- | Nucleotide metabolism |
|  | 1.7.99.4 | Nucleotide metabolism |
|  | 6.1.1.10 | Metabolism of other amino acids |
|  | 1.17.1.4 | Nucleotide metabolism |
|  | 4.1.1.31 | Carbohydrate metabolism |
|  | 3.5.1.54 | Carbohydrate metabolism |
|  | 1.2.1.31 | Carbohydrate metabolism |
|  | 1.2.1.8 | Carbohydrate metabolism |
|  | 1.2.1.3 | Carbohydrate metabolism |
|  | 4.2.1.2 | Carbohydrate metabolism |
|  | 1.13.11.5 | Nucleotide metabolism |
|  | 2.1.1.- | Metabolism of terpenoids and polyketides |
|  | 4.1.1.17 | Biosynthesis of other secondary metabolites |
|  | 2.4.2.21 | Energy metabolism |
|  | 2.4.1.18 | Biosynthesis of other secondary metabolites |
|  | 3.2.1.21 | Lipid metabolism |
|  | 2.3.1.51 | Carbohydrate metabolism |
|  | 1.2.1.41 | Carbohydrate metabolism |
|  | 1.1.1.284 | Carbohydrate metabolism |
|  | 1.1.1.1 | Carbohydrate metabolism |
|  | 1.14.13.- | Biosynthesis of other secondary metabolites |
|  | 2.7.7.13 | Carbohydrate metabolism |
|  | 6.4.1.1 | Carbohydrate metabolism |
|  | 4.1.1.75 | Carbohydrate metabolism |
|  | 3.6.3.30 | ATPase |
|  | 6.4.1.5 | Biosynthesis of other secondary metabolites |
|  | 3.6.1.19 | Nucleotide metabolism |
|  | 4.4.1.1 | Lyase |
|  | 5.1.3.20 | Lipid metabolism |
|  | 3.4.-.- | Hydrolase |
|  | 1.10.3.- | Biosynthesis of other secondary metabolites |
|  | 5.5.1.2 | Lipid metabolism |
|  | 2.5.1.1 | Metabolism of terpenoids and polyketides |
|  | 2.5.1.10 | Metabolism of terpenoids and polyketides |
|  | 2.5.1.29 | Metabolism of terpenoids and polyketides |
|  | 1.13.11.15 | Nucleotide metabolism |
|  | 6.4.1.2 | Lipid metabolism |
|  | 6.3.4.14 | Nucleotide metabolism |
|  | 4.2.1.20 | Carbohydrate metabolism |
|  | 6.3.2.10 | Metabolism of other amino acids |
|  | 6.1.1.19 | Metabolism of other amino acids |
|  | 1.2.1.- | Mechanisms of cofactors and vitamins |
|  | 4.1.1.65 | Lipid metabolism |
|  | 1.1.1.22 | Carbohydrate metabolism |
|  | 2.3.1.9 | Lipid metabolism |
|  | 4.2.1.10 | Lipid metabolism |
|  | 3.5.4.4 | Nucleotide metabolism |
|  | 3.1.6.1 | Lipid metabolism |
|  | 2.4.99.12 | Transferase |
|  | 2.4.99.13 | Lipid metabolism |
|  | 2.4.99.14 | Transferase |
|  | 2.4.99.15 | Transferase |
|  | 2.1.2.3 | Nucleotide metabolism |
|  | 3.5.4.10 | Nucleotide metabolism |
|  | 2.7.7.60 | Metabolism of terpenoids and polyketides |
|  | 2.1.2.13 | Biosynthesis of other secondary metabolites |
|  | 1.1.1.305 | Biosynthesis of other secondary metabolites |
|  | 3.5.4.3 | Nucleotide metabolism |
|  | 6.1.1.9 | Metabolism of other amino acids |
|  | 2.1.2.1 | Biosynthesis of other secondary metabolites |
|  | 3.5.1.41 | Biosynthesis of other secondary metabolites |
|  | 3.1.11.5 | Hydrolase |
|  | 3.5.1.16 | Carbohydrate metabolism |
|  | 3.5.3.13 | Carbohydrate metabolism |
|  | 3.5.1.6 | Nucleotide metabolism |
|  | 1.1.5.4 | Carbohydrate metabolism |
|  | 2.1.1.195 | Energy metabolism |
|  | 1.7.1.4 | Nucleotide metabolism |
|  | 1.4.4.2 | Carbohydrate metabolism |
|  | 2.2.1.6 | Metabolism of other amino acids |
|  | 2.7.7.4 | Nucleotide metabolism |
|  | 2.7.1.25 | Nucleotide metabolism |
|  | 3.6.3.17 | ATPase |
|  | 2.6.99.2 | Mechanism of cofactors and vitamins |
|  | 2.4.2.14 | Nucleotide metabolism |
|  | 1.1.1.1 | Carbohydrate metabolism |
|  | 3.5.1.10 | Carbohydrate metabolism |
|  | 1.17.4.1 | Nucleotide metabolism |
|  | 1.2.1.12 | Carbohydrate metabolism |
|  | 4.1.1.44 | Lipid metabolism |
|  | 4.3.1.3 | Nucleotide metabolism |
|  | 2.5.1.- | Lipid metabolism |
|  | 2.7.7.23 | Biosynthesis of other secondary metabolites |
|  | 2.3.1.157 | Carbohydrate metabolism |
|  | 2.3.3.13 | Lipid metabolism |
|  | 3.7.1.2 | Nucleotide metabolism |
|  | 4.2.1.3 | Carbohydrate metabolism |
|  | 4.2.1.- | Nucleotide metabolism |
|  | 2.8.3.12 | Nucleotide metabolism |
|  | 6.3.5.4 | Biosynthesis of other secondary metabolites |
|  | 1.1.99.1 | Carbohydrate metabolism |
|  | 4.2.1.79 | Lipid metabolism |
|  | 1.14.13.2 | Lipid metabolism |
|  | 1.2.4.1 | Carbohydrate metabolism |
|  | 3.6.3.32 | ATPase |
|  | 2.4.1.1 | Carbohydrate metabolism |
|  | 1.6.99.3 | Energy metabolism |
|  | 3.1.3.1 | Lipid metabolism |
|  | 2.7.7.38 | Lipid metabolism |
|  | 2.8.1.7 | Mechanisms of cofactors and vitamins |
|  | 2.7.8.- | Lipid metabolism |

**S5** - Summary of assembly data for three-way comparison of SPAdes, IDBA-UD and Velvet Optimizer, focusing on N50, number of contigs assembled and largest contiguous sequence assembled.

|  | **Pa Sample ID** | **SPAdes N50** | **SPAdes contigs > 500 bp** | **SPAdes longest contig (bp)** | **IDBA-UD N50** | **IDBA-UD contigs > 500 bp** | **IDBA-UD longest contig (bp)** | **Velvet optimizer N50** | **Velvet optimizer contigs > 500 bp** | **Velvet optimizer longest contig (bp)** |
| --- | --- | --- | --- | --- | --- | --- | --- | --- | --- | --- |
| **< 10 years since clinical diagnosis nCFBR** | **49** | 4,467 | 5 | 4,467 | 4,467 | 2 | 4,467 | 2,118 | 3 | 2,118 |
|  | **50** | 17,035 | 30 | 48,272 | 16,764 | 24 | 37,577 | 8,869 | 41 | 28,497 |
|  | **52** | 8,363 | 13 | 14,150 | 8,274 | 14 | 11,198 | 3,566 | 14 | 9,457 |
|  | **57** | 4,085 | 2 | 4,085 | 3,059 | 1 | 3,059 | 812 | 1 | 812 |
|  | **67** | 886 | 6 | 3,110 | 2,529 | 1 | 2,529 | 1,449 | 2 | 1,449 |
|  | **68** | 8,089 | 17 | 24,425 | 8,274 | 15 | 11,180 | 3,563 | 14 | 11,151 |
|  | **72** | 25,633 | 2 | 25,633 | 37,350 | 1 | 37,350 | 32,516 | 2 | 32,516 |
|  | **73** | 2,257 | 41 | 6,582 | 5,815 | 15 | 19,653 | 3,702 | 16 | 15,980 |
|  | **74** | 8,237 | 11 | 14,045 | 8,307 | 14 | 11,163 | 4,194 | 19 | 9,496 |
|  | **76** | 19,009 | 21 | 35,341 | 18,969 | 10 | 35,340 | 18,985 | 13 | 31,565 |
|  | **77** | 61,772 | 1 | 61,772 | 61,570 | 1 | 61,570 | 25,201 | 3 | 26,529 |
|  | **80** | 8,122 | 13 | 25,526 | 8,307 | 14 | 10,037 | 3,497 | 14 | 8,943 |
|  | **81** | 649 | 2 | 649 | N/A | N/A | N/A | N/A | N/A | N/A |
|  | **82** | 2,757 | 6 | 4,408 | 4,798 | 2 | 4,798 | 2,671 | 8 | 2,713 |
|  | **86** | 8,363 | 10 | 27,106 | 8,307 | 14 | 11,122 | 3,979 | 16 | 11,175 |
|  | **91** | 61,772 | 1 | 61,772 | 29,967 | 3 | 30,772 | 25,193 | 3 | 26,515 |
|  | **94** | 882 | 3 | 1,348 | 1,323 | 1 | 1,323 | 575 | 2 | 575 |
|  |  |  |  |  |  |  |  |  |  |  |
| **> 10 years since clinical diagnosis nCFBR** | **46** | 10,760 | 17 | 36,835 | 10,250 | 7 | 28,792 | 9,991 | 17 | 36,766 |
|  | **47** | 15,903 | 29 | 38,353 | 15,698 | 26 | 38,341 | 15,686 | 40 | 38,341 |
|  | **48** | 3,385 | 16 | 5,970 | 39,556 | 1 | 39,556 | N/A | N/A | N/A |
|  | **51** | 26,325 | 26 | 53,682 | 15,721 | 29 | 38,116 | 14,917 | 44 | 38,255 |
|  | **53** | 50,013 | 1 | 50,013 | 49,807 | 1 | 49,807 | 41,926 | 2 | 41,926 |
|  | **54** | 21,563 | 37 | 41,593 | 17,868 | 46 | 40,398 | 19,243 | 49 | 40,329 |
|  | **55** | 14,052 | 14 | 37,359 | 8,786 | 16 | 37,021 | 4,881 | 20 | 37,204 |
|  | **56** | 17,035 | 49 | 38,328 | 17,868 | 49 | 38,316 | 13,241 | 60 | 38,328 |
|  | **58** | 3,032 | 8 | 3,249 | 3,032 | 4 | 3,249 | 900 | 9 | 3,375 |
|  | **59** | 61,772 | 1 | 61,772 | 61,582 | 1 | 61,582 | 30,843 | 4 | 30,843 |
|  | **60** | 9,128 | 6 | 9,128 | 10,076 | 1 | 10,076 | 2,595 | 8 | 4,556 |
|  | **61** | 8,089 | 14 | 25,388 | 11,167 | 17 | 11,167 | 3,729 | 17 | 11,209 |
|  | **62** | 40,441 | 2 | 40,441 | 40,352 | 2 | 40,352 | 40,414 | 7 | 40,414 |
|  | **63** | 37,715 | 1 | 37,715 | 20,272 | 2 | 20,272 | 19,523 | 5 | 19,523 |
|  | **64** | 61,772 | 1 | 61,772 | 61,609 | 1 | 61,609 | 12,408 | 6 | 17,991 |
|  | **65** | 1,193 | 57 | 5,705 | 4,071 | 17 | 11,107 | 3,760 | 16 | 9,306 |
|  | **66** | N/A | N/A | N/A | N/A | N/A | N/A | N/A | N/A | N/A |
|  | **69** | 31,654 | 26 | 90,429 | 31,625 | 26 | 37,639 | 9,645 | 38 | 31,555 |
|  | **70** | 3,712 | 7 | 4,114 | 5,574 | 2 | 5,574 | 806 | 9 | 1,065 |
|  | **71** | 594 | 1 | 594 | N/A | N/A | N/A | N/A | N/A | N/A |
|  | **75** | 37,660 | 11 | 52,654 | 37,510 | 9 | 52,534 | 20,956 | 21 | 31,826 |
|  | **78** | 1,806 | 45 | 6,512 | 5,784 | 18 | 9,892 | 4,009 | 16 | 10,929 |
|  | **79** | 61,772 | 1 | 61,772 | 61,609 | 1 | 61,609 | 6,092 | 10 | 19,856 |
|  | **85** | 12,853 | 43 | 34,949 | 14,670 | 55 | 33,749 | 10,508 | 56 | 33,590 |
|  | **87** | 1,608 | 17 | 2,327 | 1,690 | 12 | 2,287 | 1,523 | 17 | 2,287 |
|  | **88** | 26,228 | 32 | 38,158 | 17,717 | 42 | 37,876 | 13,160 | 40 | 26,010 |
|  | **89** | 8,363 | 13 | 22,288 | 4,905 | 19 | 9,710 | 1,877 | 25 | 3,681 |
|  | **90** | N/A | N/A | N/A | N/A | N/A | N/A | N/A | N/A | N/A |
|  | **92** | 61,772 | 1 | 61,772 | 44,032 | 2 | 44,032 | 31,735 | 3 | 31,735 |
|  | **93** | 10,045 | 10 | 15,044 | 7,128 | 8 | 23,183 | 5,913 | 11 | 11,529 |
|  |  |  |  |  |  |  |  |  |  |  |
| **Pediatric CF** | **13** | 8,330 | 17 | 20,288 | 8,307 | 13 | 20,467 | 4,878 | 14 | 15,649 |
|  | **14** | 17,035 | 41 | 38,360 | 17,717 | 46 | 38,360 | 13,243 | 58 | 38,356 |
|  | **22** | 61,772 | 7 | 61,772 | 21,201 | 5 | 26,599 | 1,364 | 50 | 2,323 |
|  | **23** | 4,681 | 21 | 8,473 | 4,765 | 11 | 8,442 | 4,666 | 21 | 8,463 |
|  | **32** | 17,035 | 43 | 32,899 | 17,558 | 48 | 30,584 | 8,268 | 60 | 31,650 |
|  | **40** | 2,214 | 42 | 7,645 | 5,815 | 15 | 12,799 | 3,523 | 16 | 8,460 |
|  | **43** | 3,853 | 21 | 6,849 | 2,942 | 18 | 7,317 | 3,985 | 19 | 9,873 |
|  | **44** | 52,658 | 2 | 52,658 | 37,577 | 4 | 37,832 | 31,041 | 3 | 37,504 |
|  | **45** | 32,297 | 16 | 38,297 | 38,286 | 4 | 38,386 | 38,284 | 17 | 38,284 |
|  | **83** | 2,747 | 27 | 5,652 | 3,247 | 12 | 5,624 | 1,077 | 25 | 2,160 |
|  |  |  |  |  |  |  |  |  |  |  |
| **Adult CF** | **1** | 8,330 | 14 | 25,454 | 8,274 | 15 | 11,253 | 4,554 | 15 | 11,351 |
|  | **2** | 2,064 | 43 | 3,807 | 2,308 | 28 | 3,802 | 792 | 36 | 1,718 |
|  | **3** | 8,039 | 11 | 27,661 | 2,942 | 20 | 7,053 | 3,658 | 18 | 9,286 |
|  | **4** | 5,714 | 30 | 16,984 | 5,713 | 27 | 16,937 | 760 | 26 | 1,239 |
|  | **5** | 52,800 | 2 | 52,800 | 37,577 | 3 | 40,523 | 28,349 | 6 | 37,510 |
|  | **6** | 61,649 | 3 | 61,649 | 57,517 | 4 | 57,417 | 57,714 | 10 | 57,714 |
|  | **7** | 14,035 | 14 | 37,361 | 37,280 | 18 | 37,280 | 11,114 | 18 | 37,204 |
|  | **8** | 1,909 | 49 | 5,871 | 11,439 | 17 | 11,439 | 3,569 | 17 | 11,176 |
|  | **9** | 8,363 | 15 | 24,014 | 8,274 | 14 | 11,803 | 3,560 | 12 | 9,299 |
|  | **10** | 9,016 | 14 | 20,434 | 7,726 | 6 | 20,386 | 1,677 | 28 | 5,159 |
|  | **11** | 1,606 | 8 | 2,802 | 4,822 | 3 | 4,822 | 1,519 | 8 | 2,515 |
|  | **12** | 3,603 | 29 | 7,027 | 3,646 | 22 | 6,696 | 3,521 | 29 | 7,017 |
|  | **15** | 62,077 | 6 | 62,077 | 36,064 | 8 | 37,632 | 10,313 | 17 | 20,962 |
|  | **16** | 1,670 | 46 | 7,099 | 5,161 | 17 | 11,467 | 3,525 | 18 | 9,331 |
|  | **17** | 52,664 | 2 | 52,664 | 52,623 | 2 | 52,623 | 28,259 | 3 | 37,474 |
|  | **18** | 1,366 | 63 | 4,913 | 4,698 | 16 | 8,307 | 2,021 | 22 | 4,868 |
|  | **19** | 23,619 | 4 | 29,993 | 20,028 | 6 | 28,817 | N/A | N/A | N/A |
|  | **20** | 61,562 | 6 | 61,562 | 34,762 | 7 | 37,677 | 25,865 | 16 | 37,420 |
|  | **21** | 3,055 | 20 | 11,703 | 8,274 | 15 | 9,366 | 9,300 | 14 | 11,150 |
|  | **24** | 4,019 | 6 | 4,019 | 3,822 | 1 | 3,822 | 794 | 8 | 1,864 |
|  | **25** | 10,598 | 28 | 44,244 | 9,896 | 27 | 37,577 | 10,341 | 38 | 24,798 |
|  | **26** | 37,617 | 17 | 41,525 | 24,044 | 12 | 37,559 | 10,208 | 21 | 37,479 |
|  | **27** | 39,705 | 1 | 39,705 | 39,579 | 1 | 39,579 | 39,558 | 1 | 39,558 |
|  | **28** | 3,772 | 24 | 7,654 | 8,274 | 13 | 19,484 | N/A | N/A | N/A |
|  | **29** | 10,321 | 50 | 28,830 | 13,226 | 53 | 27,631 | 12,607 | 54 | 27,562 |
|  | **30** | 11,205 | 58 | 33,124 | 7,741 | 64 | 37,496 | 5,673 | 85 | 27,617 |
|  | **31** | 26,208 | 37 | 80,678 | 18,705 | 31 | 70,475 | 15,245 | 49 | 67,147 |
|  | **33** | 39,659 | 1 | 39,659 | 25,118 | 2 | 25,118 | 19,927 | 2 | 19,927 |
|  | **34** | 61,597 | 7 | 61,597 | 23,998 | 7 | 26,599 | 1,749 | 38 | 4,913 |
|  | **35** | 17,035 | 38 | 38,239 | 17,558 | 50 | 38,047 | 11,482 | 54 | 38,244 |
|  | **36** | 37,242 | 8 | 37,654 | 25,055 | 7 | 37,576 | 28,201 | 12 | 37,474 |
|  | **37** | 8,330 | 12 | 14,034 | 8,274 | 14 | 11,163 | 3,679 | 15 | 11,087 |
|  | **38** | 3,384 | 32 | 8,039 | 4,468 | 18 | 8,007 | 1,113 | 49 | 3,288 |
|  | **39** | 2,936 | 29 | 7,842 | 5,781 | 16 | 19,353 | 3,073 | 21 | 11,263 |
|  | **41** | 12,686 | 34 | 28,104 | 9,242 | 45 | 28,071 | 6,299 | 44 | 27,593 |
|  | **42** | 28,149 | 32 | 41,380 | 26,266 | 46 | 38,243 | 27,649 | 44 | 40,129 |
|  | **84** | 28,144 | 4 | 28,144 | 28,049 | 4 | 28,049 | 23,219 | 7 | 23,219 |

S6 Command line entry for each bioinformatic package

Velvet Optimizer

shufflesequences_fastq.pl read1.fastq read2.fastq shufflesequence.fastq

Velvet Optimizer v 2.2.5 was used with Velvet v 1.2.10 to assemble metagenomes. The assembled contigs were used with GeneWise2 to identify putative Ig-like domains. The command used to assemble with VelvetOptimizer was velvetoptimizer.pl –f ‘-fastq read.fastq’ –t 4.

>load-into-counting.py -N 4 -X 4E9 out.kh shufflesequence.fastq
>abundance-dist.py out.kh shufflesequence.fastq out.hist

SPAdes

The command used to assemble the phage using SPAdes was spades.py –k 21, 33, 55, 77, 99, 127 –careful –s /read.fastq –o /spades.out.

IDBA-UD

Prior to building IDBA-UD we changed the short_sequence.h file from kMaxShortSequence = 128 to 250. Using the IDBA-UD assembler the following parameter we used idba_ud.py –l /read.fasta –o/idba.out --min_contigs 500.
